# Integrated network pharmacology and experimental verification to reveal the role of Shezhi Huangling Decoction against glioma by inactivating PI3K/Akt-HIF1A axis

**DOI:** 10.1016/j.heliyon.2024.e34215

**Published:** 2024-07-06

**Authors:** Xiaobing Zhang, Xian Shao, Qingquan Bao, Lingyan He, Xuchen Qi

**Affiliations:** a Department of Neurosurgery, Sir Run Run Shaw Hospital, Zhejiang University School of Medicine, Hangzhou, Zhejiang, China; b Department of Neurosurgery, Shaoxing People's Hospital, Shaoxing, Zhejiang, China; c Department of Medical Research Center, Shaoxing People's Hospital, Shaoxing, Zhejiang, China; d Department of Traditional Chinese Medicine, Shaoxing People's Hospital, Shaoxing, Zhejiang, China

**Keywords:** Shezhi Huangling, Immune, Network pharmacology, Glioma

## Abstract

Shezhi Huangling Decoction (SHD) has been proven clinically effective in regulating metabolic and immune homeostasis in the treatment of glioma. The investigation aimed to deconstruct the active constituents and mechanisms of SHD. Effects of SHD on malignant characteristics of HS683 and KNS89 cells have been investigated by CCK-8, clone formation, flow cytometry, and Transwell assays. A mouse xenograft model was established to assess the effect of SHD or SHD + temozolomide (TMZ) *in vivo*. A total of 461 constituents were found from SHD in UPLC/Q-TOF-MS/MS analysis. Functional enrichment analysis showed that pathway in cancer, proteoglycans in cancer, regulation of epithelial cell proliferation, inflammation/immune, gliogenesis, brain development, cell adhesion, and autophagy could participate in the treatment of SHD. Additionally, 9 hub genes (AKT1, TP53, CTNNB1, STAT3, EGFR, VEGFA, PIK3CA, ERBB2, and HIF1A) were identified as hub genes. Moreover, we found that SHD may greatly reduce the migration and accelerate apoptosis of HS683 and KNS89 cells. Additionally, SHD coordinates TMZ to restrict tumor growth were found in the mice. Our results suggest that the malignant behaviors of glioma cells are suppressed by SHD and the mechanism may be closing on the inhibition of the PI3K/Akt-HIF1A axis. SHD may serve as a synergistic therapeutic choice for TMZ to suppress glioblastoma growth.

## Highlights

• AKT1, TP53, CTNNB1, STAT3, EGFR, VEGFA, PIK3CA, ERBB2, and HIF1A may serve as potential targets of SHD.
• SHD significantly inhibited the malignant phenotypes of HS683 and KNS89 cells.
• SHD cooperates with TMZ significantly inhibited tumor growth *in vivo*.

## 1 Introduction

Glioblastoma multiforme (GBM) is a common intracranial primary malignant tumor, which has been treated with a combination of surgery, radiotherapy, and immunotherapy for many years [1,2]. However, there is no breakthrough in the overall treatment of brain glioma, and the search for effective treatment methods is still a hot spot in clinical research. Traditional Chinese medicine (TCM) has a long history, and more and more studies on the treatment of malignant tumors with Chinese medicine are being conducted, which has gained wide attention [3,4]. The complex composition of Chinese medicine, especially the TCM formula, could exert anti-tumor effects from multiple targets and has great potential and advantages in the treatment of malignant tumors [5,6].

The original formula of Shezhi Huangling Decoction (SHD), composed of Hedyotis Diffusae Herba, Scutellariae Barbatae Herba, Rehmanniae Radix Praeparata, Smilacis Glabrae Rhixoma, Hedysarum Multijugum Maxim, Atractylodes Macrocephala Koidz, Angelicae Sinensis Radix, Ficus carica, Coptidis Rhizoma, Radix Rhei Et Rhizome, Radix Puerariae, and licorice, was initially employed and developed by professor Jiang et al. in Affiliated Hospital of Shandong University of Traditional Chinese Medicine according to the syndrome differentiation of Zang-fu viscera [7,8]. It has been demonstrated that combination therapy with SHD and bis-chloroethylnitrosourea (BCNU) dramatically boosts the patient's functional status, medication effectiveness, and survival rate when compared to BCNU alone [7]. Furthermore, Liu et al. reported that SHD significantly inhibited the proliferation, migration and invasion via up-regulation of miR-1298-5p and inhibition of TGIF1 in U87 and U251 cells [9]. However, as a TCM formula, SHD is characterized by complex components, multiple action targets and multiple action pathways, and it is difficult to systematically and completely elucidate its mechanism of action by traditional pharmacological research methods. Network pharmacology is an important part of systems biology, and its holistic, systematic and drug-interaction-oriented characteristics are consistent with the basic characteristics of Chinese medicine, which can be used to analyze the interaction between drugs and specific nodes in the network, and is an emerging discipline to reveal the effect of TCM on the regulatory network of the organism from the systemic level, which provides a new idea to study the modern pharmacological mechanism of action of TCM [10,11]. Most importantly, on the basis of network pharmacology, a novel significant bioactive component of the TCM formula may also be discovered [12,13].

Since its approval in Europe in 1997, temozolomide (TMZ) has rapidly become the first-line alkylating agent in the treatment of glioma, and Stupp [2] published a study in 2002 showing that continuous daily oral TMZ in combination with radiotherapy had a high safety profile and was effective in prolonging the survival of GBM patients [14]. A further study by Stupp [3] in 2005 showed that radiotherapy combined with TMZ was effective in prolonging median survival in 573 patients with GBM from 85 centers, with median survival increasing from 12.1 to 14.6 months, two-year survival increasing from 10.4 % to 26.5 % compared to the radiotherapy alone group, and only 7 % of patients had grade 3–4 hematologic toxicities [15]. Nevertheless, the majority of patients don't respond to TMZ while they're receiving treatment, which is mainly due to the drug resistance of TMZ [16]. Recent studies have confirmed that TCM shows unique advantages in enhancing the sensitivity of antitumor drugs and overcoming tumor resistance [17,18], and it is of significant practical significance to further search for new drugs as sensitizers for chemotherapy drugs to provide more options for clinical treatment.

In this study, we first screened the potential active components and targets of SHD against brain tumors by ultraperformance liquid chromatography-quadrupole time-of-flight/mass spectrometry (UPLC-Q-TOF/MS) and network pharmacology analyses to reveal the "multi-component/multi-target/multi-pathway" anti-tumor mode of action of SHD, and then investigated the anti-tumor effect of SHD through biomolecular experiments. After that, BALB/c tumor-bearing nude mice were used to study the antitumor effects of SHD combined with TMZ, and then preliminarily evaluate the pharmacological potential mechanism of SHD in combination with TMZ. In general, these findings might highlight the theoretical basis of SHD for further studies on glioma treatment.

## 2 Materials and methods

### 2.1 Preparation of SHD

Hedyotis Diffusae Herba (15 g), Scutellariae Barbatae Herba (15 g), Rehmanniae Radix Praeparata (15 g), Smilacis Glabrae Rhixoma (15 g), Hedysarum Multijugum Maxim (15 g), Atractylodes Macrocephala Koidz (15 g), Angelicae Sinensis Radix (15 g), Ficus carica (9 g), Coptidis Rhizoma (9 g), Radix Rhei Et Rhizome (9 g), Radix Puerariae (9 g), and licorice (3 g) were all purchased and identified by the First Affiliated Hospital of Zhejiang Chinese Medical University (Zhejiang, China). A total of 144 g of raw SHD herbs were soaked in a 10-fold amount of distilled water for 60 min and boiled for 2h in a porcelain jar. Then, the aqueous extract was collected. Next, the herb residues were extracted again with a 5-fold amount of distilled water for 30 min. All the aqueous extract of SHD was collected and filtered by a vacuum filter and then concentrated to 0.1 g/mL and stored at 4 °C until further use.

### 2.2 Analysis of chemical components in SHD aqueous extract

Identification of chemical components in aqueous extract of SHD was performed on an ultra-performance liquid chromatography (UHPLC) system (Vanquish, Thermo Fisher Scientific) with a Waters UPLC BEH C18 column (1.7 μm*2.1*100 mm). The flow rate was set at 0.5 mL/min and the sample injection volume was set at 5 μL. The mobile phase consisted of 0.1 % formic acid in water (A) and 0.1 % formic acid in acetonitrile (B). The multi-step linear elution gradient program was as follows: 0–11 min, 85-25 % A; 11–12 min, 25-2% A; 12–14 min, 2-2% A; 14–14.1 min, 2–85 % A; 14.1–16 min, 85-85 % A. An Orbitrap Exploris 120 mass spectrometer coupled with Xcalibur software was employed to obtain the MS and MS/MS data based on the IDA acquisition mode. During each acquisition cycle, the mass range was from 100 to 1500, and the top four of every cycle were screened and the corresponding MS/MS data were further acquired. Sheath gas flow rate: 35 Arb, Aux gas flow rate: 15 Arb, Ion Transfer Tube Temp: 350 °C, Vaporizer Temp: 350 °C, Full ms resolution: 60000, MS/MS resolution: 15000, Collision energy: 16/32/48 in NCE mode, Spray Voltage: 5.5 kV (Positive) or −4 kV (Negative).

### 2.3 Pharmacokinetic evaluation of active components

The active components of SHD were also acquired and filtered out from the Traditional Chinese Medicine Systems Pharmacology Database and Analysis Platform (TCMSP, https://old.tcmsp-e.com/tcmsp.php) according to the absorption, distribution, metabolism, excretion (ADME) criteria with oral bioavailability (OB)≥30 % and drug-likeness (DL)≥0.18 [19]. Predicted targets of identified active components were retrieved from the SwissTargetPrediction databases (http://www.Swisstargetprediction.ch) [20] by using the SMILES structures of components from PubChem (https://PubChem.ncbi.nlm.nih.gov) [21]. Next, the targets were standardized in the UniProt (https://www.uniprot.org) database with species set as ‘human’ [22].

### 2.4 Collection of potential glioma-related targets

The targets associated with the glioma were acquired by searching the GeneCards (https://www.genecards.org/) [23], GenCLip 3 (http://ci.smu.edu.cn/genclip3/) [24], and OMIM (https://omim.org/search/advanced/) [25] using the key term “brain glioma”. Subsequently, a Venn diagram emerged in the online Venny 2.1 mapping platform (http://bioinfogp.cnb.csic.es/tools/venny/index.html) using the predicted targets and glioma-related datasets to obtain the overlapping genes for further analysis.

### 2.5 Enrichment analysis and network construction

The gene ontology (GO) and Kyoto Encyclopedia of Genes and Genomes (KEGG) pathway enrichment analysis were performed using the Metascape (http://metascape.org/) [26] with *P-value* set as “*P < 0.01*” and species set as “Homo sapiens”. Then, the results of the top 20 terms were colored by -log10 (*P-values*) and illustrated in a bar chart. To further excavate the hub target genes, a protein-protein interaction (PPI) network was conducted by Search Tool for the Retrieval of Interacting Genes (STRING, https://www.string-db.org/) [27] database with default parameters. Following, the PPI network was visualized in the Cytoscape software (v3.7.2, https://cytoscape.org/) [28,29]. Moreover, the NetworkAnalyzer plugin for Cytoscape was used to calculate the topological parameters of nodes such as betweenness centrality, closeness centrality, and degree value. After that, the top 9 nodes were recognized as hub genes with degree value higher than the 1.5 time homologous median values in the PPI network. Additionally, the “herb-component” network and “overlapping genes-component” network were constructed using Cytoscape to systematically investigate the underlying material base of SHD in the treatment of glioma.

### 2.6 Expression levels and prognostic value analysis of overlapping genes

The Gene Expression Profiling Interactive Analysis (GEPIA2, http://gepia2.cancer-pku.cn/#index) database was used to assess expression levels of hub genes between cancers and paired normal tissue in GBM and Lower-grade glioma (LGG) patients [30]. Furthermore, we used The University of ALabama at Birmingham CANcer data analysis Portal (UALCAN, http://ualcan.path.uab.edu/analysis.html) to comprehend the relationship between hub gene expression and overall survival (OS) in GBM and LGG patients [31].

### 2.7 Cell culture

Human glioblastoma cell lines HS683 and KNS89 were purchased from iCell Bioscience Inc (Shanghai, China) and grown in Dulbecco's Modified Eagle Medium (DMEM, REF12800-017, Gibco, USA) containing 10 % fetal bovine serum (FBS, FS301-02, TRANSGEN, Beijing, China) and 1 % penicillin/streptomycin (Gibco, USA). All cells were cultivated at 37 °C with 5 % CO_2_ in a saturated humidity incubator (BB150, Thermo Scientific).

### 2.8 Cell counting Kit-8 (CCK-8) assay

The 3rd generation glioma HS683 and KNS89 cells in the logarithmic growth period were collected in 15 mL centrifuge tubes after being centrifuged, washed, diluted, and counted. Then, the cell concentration was adjusted to 6 × 10^4^/mL and inoculated into 96-well plates at 100 μL/well in a CO_2_ incubator overnight. Afterwards, the cells were treated with SHD at different doses of 0, 5, 10, 20, 40, and 80 μg/mL, respectively. After incubation for 24, 48, and 72 h, the 96-well plates were incubated with CCK-8 solution at 10 μL/well and incubated for 4 h. After that, The absorbance at a wavelength of 450 nm was assessed by utilizing a microplate reader (CMaxPlus, Molecular Devices), and the cell viability was calculated.

### 2.9 Colony formation assay

HS683 and KNS89 cells were sown in 6-well plates with 2 mL at 500 cells/1 ml medium/well and incubated at 37 °C for 24 h. Then, the HS683 and KNS89 cells were treated with SHD (5, 10, 20 μg/mL) for 10 days for colony growth. Following the removal of the media, the HS683 and KNS89 cells were fixed with 4 % paraformaldehyde (P804536, Macklin, Shanghai, China) for 60 min at room temperature, and stained with 0.1 % crystal violet for 30 min. After washing with water and drying, photographs were taken for counting under an inverted microscope (AE2000, Motic, Xiamen, Fujian, China).

### 2.10 Cell apoptosis assay

HS683 and KNS89 cells were seeded in a 6-well plate and cultured overnight in an incubator. After incubation with SHD (5, 10, 20 μg/mL) for 48 h, the cells were treated with trypsin, washed with PBS, and resuspended in 100 μL binding buffer at a density of 1.0 × 10^6^ cells per mL. After that, HS683 and KNS89 cells were incubated with 5 μl of Annexin V-FITC and 10 μl of propidium iodide (#556547, BD Pharmingen, USA) in the dark at room temperature for 15 min and then measured with a NovoCyte flow cytometer (Agilent, California, USA). The proportion of apoptotic HS683 and KNS89 cells was analyzed with FlowJo software.

### 2.11 Transwell assay

The migration and invasion capabilities of treated HS683 and KNS89 cells were estimated by Transwell assays. For the invasion assay, Matrigel Basement Membrane Matrix (#354234, Corning, Inc., USA) was diluted at 1:8 with a serum-free medium. 30 μl of matrigel gel was added to the bottom of the upper transwell chamber. For the migration and invasion assays, HS683 and KNS89 cells in the logarithmic phase were collected and resuspended with DMEM containing different concentrations of SHD (5, 10, 20 μg/mL). Then, 5 × 10^5^ HS683 and KNS89 cells in 200 μl of serum-free DMEM were added to the upper chamber (Corning, Inc., USA), and 800 μl of DMEM supplemented with 20 % FBS was added into the lower chamber. After incubation for 48h, the cells were washed with PBS, fixed with 4 % paraformaldehyde for 30 min, stained with 0.1 % crystal violet for 30 min at room temperature, and counted under an inverted microscope. Experiments were repeated six times.

### 2.12 Animal and SHD administration

A total of 32 BALB/c-nu nude mice aged 5–6 weeks (17∼20g) were purchased from Shanghai Laboratory Animal Research Center (No. SCXK(Hu) 2017-0005, Shanghai, China). The mice were housed in the Animal Experimental Center of Zhejiang Eyong Biotechnological Co., Ltd (No. SYXK (Zhe) 2021-0033, Zhejiang, China) under standardized specific pathogen-free barrier environments with a 12 h/12 h light-dark cycle and fed with a standard diet and water ad libitum at (22 ± 2) °C, and 50–60 % relative humidity. After adaptation for 1 week, 5 × 10^6^ HS683 cells were subcutaneously injected into the rear flank of each nude mouse. As the subcutaneous transplant average volume increased to 150 mm^3^, all mice were randomly divided into 4 groups: model group, SHD group, TMZ group, and SHD + TMZ group with 8 nude mice per group. The dose of SHD in mice was calculated according to the body surface area between humans and mice with the human clinical dose of SHD. In the SHD group, mice were intragastrically treated with SHD at 22 mg/kg/twice a day for 30 days. The mice in the TMZ group applied intraperitoneal injection of 25 mg/kg TMZ once a day for 30 consecutive days. Mice in the SHD + TMZ group were daily treated with 22 mg/kg SHD plus 25 mg/kg TMZ, respectively. Tumor sizes were measured every 5 days using the following formula: volume (mm^3^) = length × width^2^/2. At the end of the experiment, the mice were sacrificed by persistent inhalation with 50 % CO_2_ for 5 min. Then, the tumor tissues used for hematoxylin-eosin (HE) staining, immunohistochemistry (IHC) and Western blot analysis were quickly removed, washed, photographed, and weighed on electronic balances.

### 2.13 HE and IHC analysis

Tumor tissue samples were fixed with 4 % paraformaldehyde for 24h at room temperature and embedded in paraffin. Next, the paraffin sections with 4-μm-thick were prepared using an RM2235 microtome (Leica, Germany), and the sections were dewaxed. After that, the sections were stained with hematoxylin and eosin (Sigma Aldrich, St. Louis, MI, USA). For IHC analysis, the sections were deparaffinized, antigen recovered, and blocked in 5 % FBS for 1h. Whereafter, the sections were incubated with primary antibody for Ki67 (1:800, # 9449S, Abcam) overnight at 4 °C, followed by HRP-labeled secondary antibody (1:5000, ab97080, Abcam). After that, the streptavidin-alkaline phosphatase solution was added for reaction, diaminobenzidine for color development, and bluing reagent counterstaining. Finally, the sections were imaged using an optical microscope and the integrated optical density was quantified using Image-Pro Plus 6.0 software (National Institutes of Health).

### 2.14 Western blot analysis

Total protein in treated HS683 and KNS89 cells and tumor tissues was extracted using the ice-cold RIPA lysis buffer (P0013B, Beyotime, China) containing protease inhibitor (60237, Beijing ComWin Biotech Co., Ltd. China). Next, the protein concentration was quantified by the BCA protein assay kit (PC0020, Solarbio, China). An equal amount of protein samples (30 μg) were separated through 10 % or 12 % sodium dodecyl sulfate-polyacrylamide gels and then transferred to a PVDF membrane (IPVH00010, Millipore, Germany). After blocking with 5 % nonfat milk for 2h, the membranes were incubated with primary antibodies against Bax (1:1000, #2774S, Cell Signaling Technologies (CST), Danvers, MA), Bcl-2 (1:1000, #15071S, CST), Caspase-3 (1:1000, #9662S, CST), E-cadherin (1:5000, #14472S, CST), Vimentin (1:1000, #5741S, CST), PIK3CA (1:1000, #4249S, CST), AKT1 (1:1000, #75692S, CST), TP53 (1:1000, #2527S, CST), CTNNB1 (1:1000, #9562S, CST), STAT3 (1:1000, #9139S, CST), EGFR (1:1000, #54359S, CST), VEGFA (1:1000, #65373S, CST), ERBB2 (1:1000, #4290S, CST), HIF1A (1:1000, #36169S), and β-actin (1:10000, ab6276, Abcam) overnight at 4 °C. The membranes were then incubated with HRP-linked anti-rabbit IgG (1:6000, #7074, CST) or anti-mouse IgG (1:6000, #7076, CST) secondary antibodies for 1h. Finally, the protein bands were developed with a chemiluminescent (ECL) Kit (P0018FS, Beyotime, China) in a chemiluminescence detection system (Clinx Science Instruments Co., Ltd, Shanghai, China) and bands were quantified using Image J software.

### 2.15 Statistical analysis

Data were expressed as means± standard deviations (SD). Statistical analyses were performed using SPSS software (v19.0, Armonk city, New York, USA). Difference between groups was analyzed by a two-tailed Student's t-test between the SHD groups and the control group. The statistical significance cutoff was set at *P < 0.05*.

## 3 Results

### 3.1 Identification of the chemical compounds in SHD

**Fig. 1:**
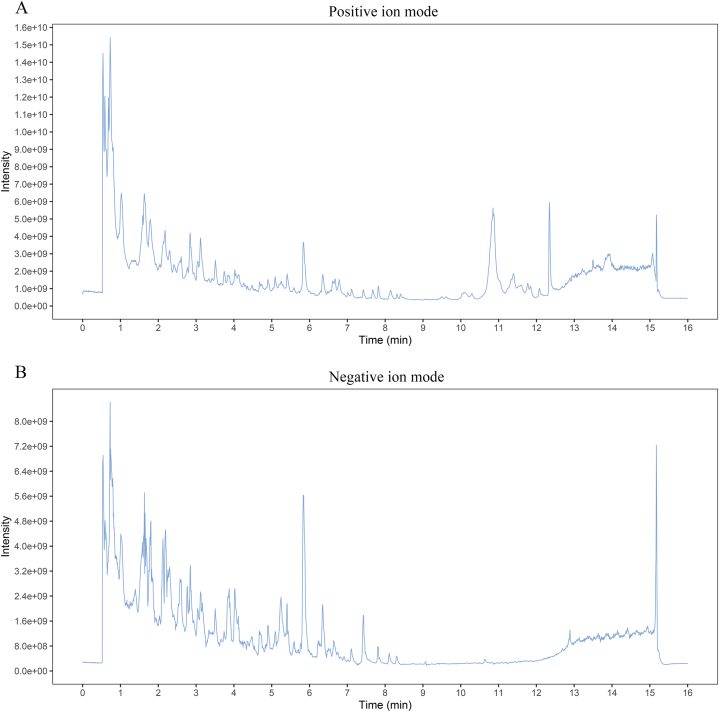
**The total ion chromatograms of the aqueous extract of SHD under the positive ion mode (A) and the negative ion mode (B)**.

**Fig. 2:**
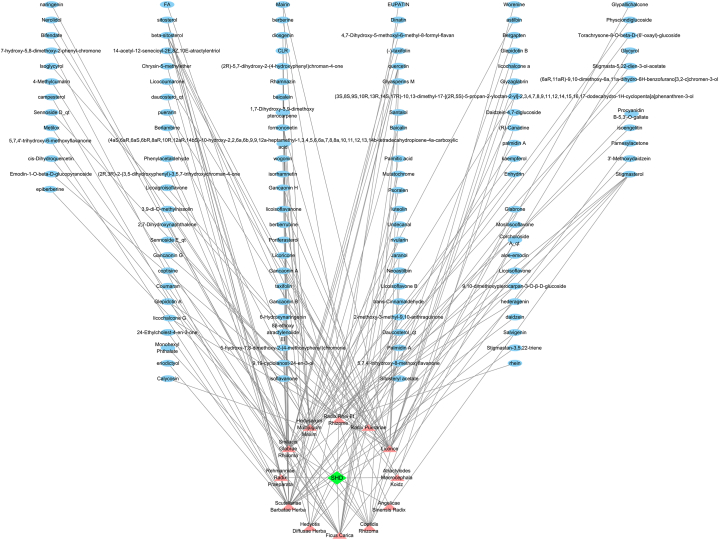
**“SHD-compound”**. The pink triangles represent the herbal medicines of SHD and the ellipses represent active compounds. (For interpretation of the references to color in this figure legend, the reader is referred to the Web version of this article.)

At first, the UHPLC-QE-MS analysis was selected to explore the potential chemical compounds in SHD. A classical total ion chromatograms of chemical compounds in SHD are illustrated in Fig. 1 with the positive ion mode (Fig. 1A) and the negative ion mode (Fig. 1B). A total of 296 compounds were identified using literature comparison in the positive ion mode, including 75 terpenoids, 60 flavonoids, 31 phenylpropanoids, 17 phenols, 16 alkaloids, and other compounds (Table S1). Similarly, a total of 165 compounds were identified in the negative ion mode, including 57 flavones, 27 terpenoids, 23 phenylpropanoids, 17 phenols, 6 aliphatic acyl, 5 quinones, 5 organic acids and derivatives, 4 aromaticity, and other compounds (Table S2). These active compounds may serve as the structural underpinning for the synergistic interaction of several components of SHD, opening the door to additional investigation. Additionally, a total of 139 identified active components of SHD were retrieved from TCMSP, 7 in Hedyotis Diffusae Herba, 29 in Scutellariae Barbatae Herba, 2 in Rehmanniae Radix Praeparata, 15 in Smilacis Glabrae Rhixoma, 16 in Hedysarum Multijugum Maxim, 3 in Atractylodes Macrocephala Koidz, 2 in Angelicae Sinensis Radix, 14 in Ficus carica, 10 in Coptidis Rhizoma, 13 in Radix Rhei Et Rhizome, 6 in Radix Puerariae, and 22 in licorice, as shown in Fig. 2. In this study, the active compounds that were in line with the ADME criteria were further used for the following network pharmacology analysis.

### 3.2 Target prediction and analysis

**Fig. 3:**
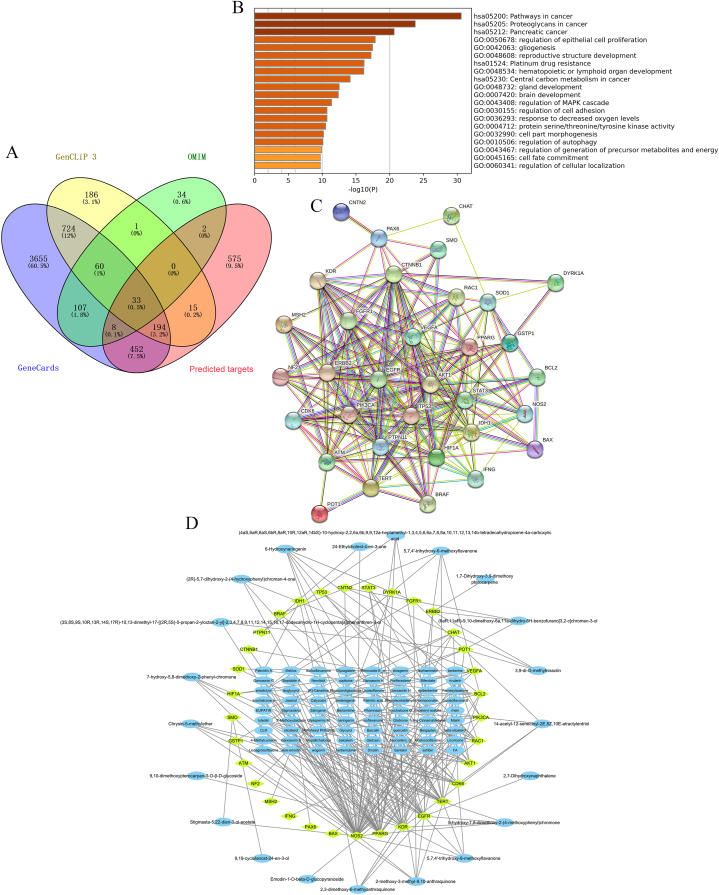
**Gene ontology (GO) enrichment analysis and construction of PPI network and “Compound-target” network for overlapping genes**. (A) Venn diagram of overlapping genes. Venn diagram generated from Venny 2.1database by using brain glioma-related target genes and predicted target genes. (B) GO analysis of overlapping genes determined by Metascape database. The X-axis shows the -log10 (P-value) of each GO term; the Y- axis shows the main biological processes and pathways. (C) PPI network of overlapping genes generated from the STRING database. (D) “Compound-target” network. The yellow prisms represent the overlapping genes and the blue ellipses represent active compounds. (For interpretation of the references to color in this figure legend, the reader is referred to the Web version of this article.)

In this study, 1279 putative targets of SHD were identified through the swisstargetprediction database after removing duplicate targets. Also, we exhaustively searched GeneCards, GenCLip, and OMIM using “brain glioma” as a keyword and identified a total of 5,233, 1,213, and 245 glioma-related target genes, respectively. Following a comparison of 1279 putative targets of SHD and glioma-related target genes, 33 overlapping genes were intersected for succedent work (Fig. 3A), as shown in Table S3. To further understand the probable mechanisms of SHD, functional enrichment analysis was carried out using the 33 overlapping genes of SHD in glioma. The results showed that the overlapping genes were mainly enriched in pathway in cancer, proteoglycans in cancer, regulation of epithelial cell proliferation, gliogenesis, brain development, cell adhesion, autophagy, and cellular localization (Fig. 3B). Meanwhile, the PPI network with 33 nodes and 220 edges based on the STRING database demonstrated the intricate relationships on the proteins encoded by these overlapping genes (Fig. 3C). For topology analysis, the findings were transferred into the Cytoscape software. The result from Table S4 shows that the hub target proteins with high betweenness centrality, closeness centrality, and degree values closer to the center include serine-threonine protein kinase (AKT1), tumor suppressor P53 (TP53), catenin beta 1 (CTNNB1), signal transducer and activator of transcription 3 (STAT3), epidermal growth factor (EGFR), vascular endothelial growth factor A (VEGFA), phosphatidylinositol-4, 5-bisphosphate 3-kinase catalytic subunit alpha (PIK3CA), epidermal growth factor receptor 2 (ERBB2, (also known as Human epidermal growth factor receptor 2 gene, HER2)), and Hypoxia inducible factor 1 alpha (HIF1A). These results suggested that the hub genes AKT1, TP53, CTNNB1, STAT3, EGFR, VEGFA, PIK3CA, ERBB2, and HIF1A may show a potential involvement in the treatment of glioma with SHD.

### 3.3 Compounds-target network analysis

To recognize the interaction between compounds and hub genes, a compounds-target network was constructed. Compounds-target network of SHD included 126 nodes (33 overlapping gene nodes, 93 compound nodes) and 246 edges, as demonstrated in Fig. 3D. Among them, EGFR with the highest degree value had 20 connections with compound nodes; Besides, AKT1, PIK3CA, and VEGFA were the top five in the degree value, which could interact with 10, 8, and 3 compounds, respectively (Table S5).

### 3.4 Expression levels and prognostic value of hub genes

**Fig. 4:**
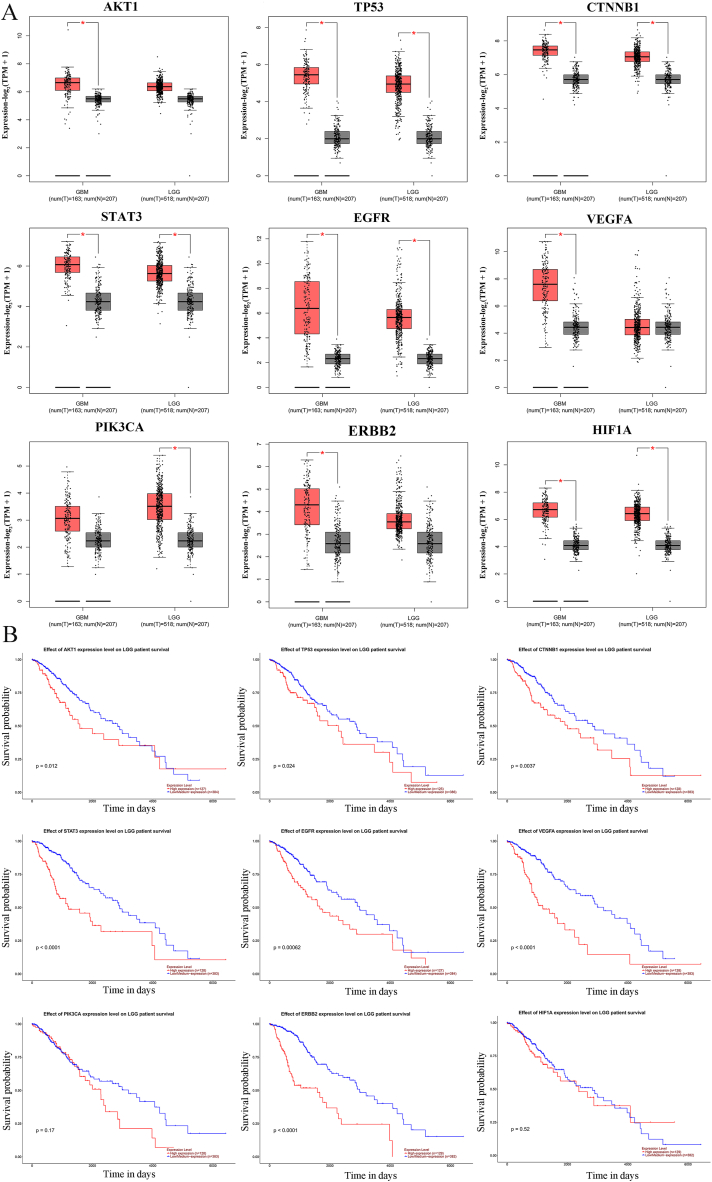
**The expression of overlapping genes in GBM and LGG and prognostic value of overlapping genes in LGG**. The expression of (A) AKT1, TP53, CTNNB1, STAT3, EGFR, VEGFA, PIK3CA, ERBB2, and HIF1A in GBM and LGG was investigated using paired tumor/normal samples from TCGA and GTEx datasets in GEPIA database. AKT1, TP53, CTNNB1, STAT3, EGFR, VEGFA, PIK3CA, ERBB2, and HIF1A were significantly highly expressed in GBM and LGG tissues. *P < 0.05. (B) Overall survival analyses of AKT1, TP53, CTNNB1, STAT3, EGFR, VEGFA, PIK3CA, ERBB2, and HIF1A in LGG cancer tissues compared with normal tissues in the UALCAN database.

To comprehensively understand the characteristics of AKT1, TP53, CTNNB1, STAT3, EGFR, VEGFA, PIK3CA, ERBB2, and HIF1A, the expression levels and prognostic value of the hub genes were assessed using the GEPIA and UALCAN databases. As demonstrated in Fig. 4A, we found that AKT1, TP53, CTNNB1, STAT3, EGFR, VEGFA, PIK3CA, ERBB2, and HIF1A were strongly expressed in GBM and LGG, particularly in GBM. We also discovered that high mRNA expression levels of AKT1, TP53, CTNNB1, STAT3, EGFR, VEGFA, and ERBB2 were associated with prominently shorter OS of LGG patients (Fig. 4B). Unexpectedly, there were no significant differences in hub genes on OS in GBM patients (data not shown) in the UALCAN database. Even so, our findings indicated that poor OS of glioma patients might relate to the high expression of hub genes in this study.

### 3.5 Effect of SHD on the malignant characteristics in HS683 and KNS89 cells

**Fig. 5:**
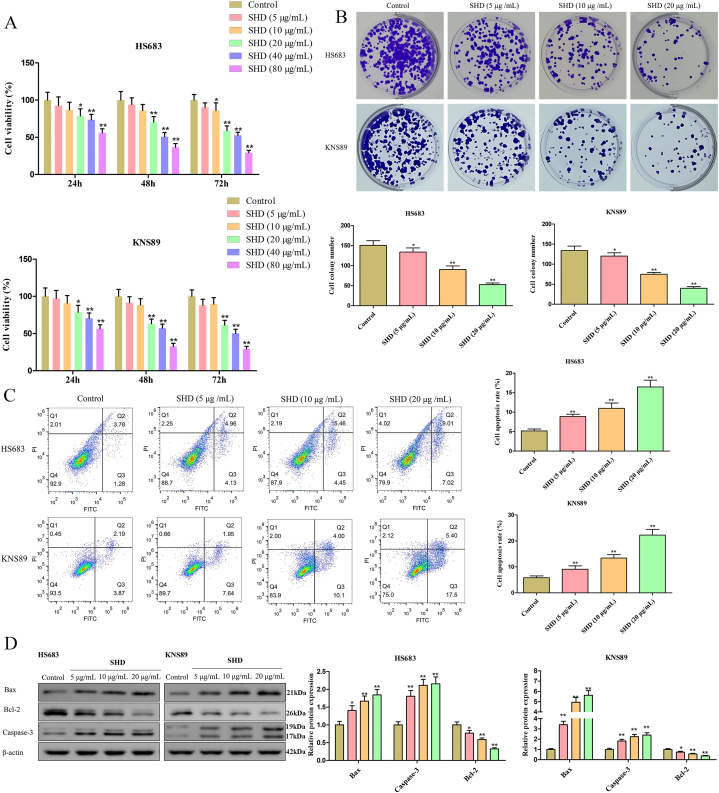
**SHD suppressed cell proliferation and promoted apoptosis of HS683 and KNS89 cells in vitro**. Cell proliferation of SHD-treated HS683 and KNS89 cells was measured by adopting (A) CCK-8 and (B) clone formation assays. (C) Apoptosis of HS683 and KNS89 cells was detected by flow cytometry. (D) Protein expression levels of apoptosis-related targets Bax, Bcl-2, and Caspase-3 were evaluated using the Western blot assay. The unedited images are referenced in Fig. S1. *P < 0.05, **P < 0.01 vs control group.

**Fig. 6:**
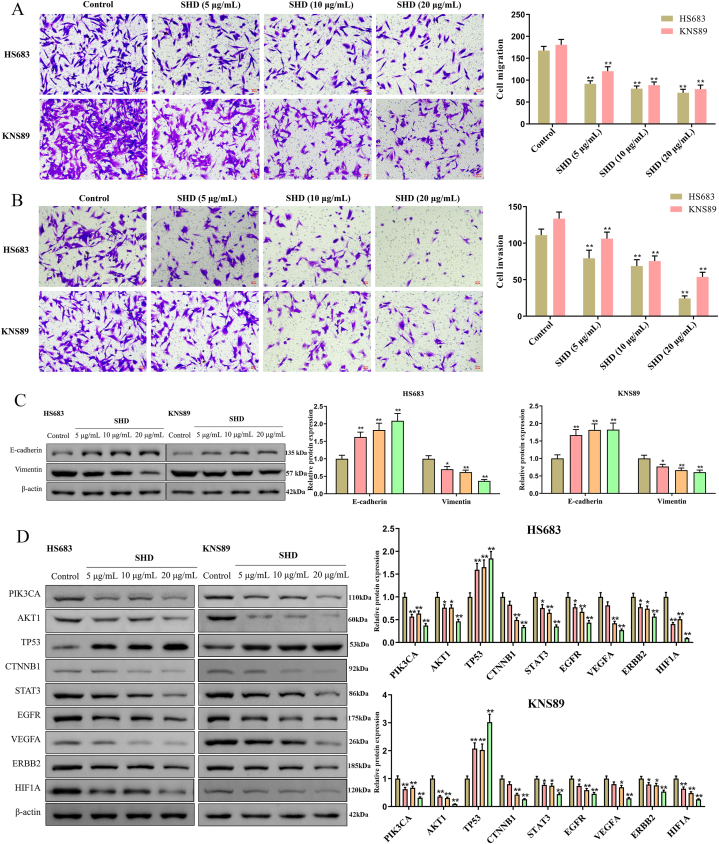
**SHD inhibited the migration, invasion and overlapping genes in HS683 and KNS89 cells**. (A) Cell migration and (B) invasion were detected in HS683 and KNS89 cells treated with SHD by transwell assay. (C–D) Protein expression levels of epithelial mesenchymal transition (EMT)-related genes (E-cadherin and Vimentin) and overlapping genes (AKT1, TP53, CTNNB1, STAT3, EGFR, VEGFA, PIK3CA, ERBB2, and HIF1A) were assessed using the Western blot assay. The unedited images are referenced in Fig. S2 and Fig. S3, respectively. *P < 0.05, **P < 0.01 vs control group.

To elucidate the influence of SHD on the cell proliferation and apoptosis of HS683 and KNS89 cells, we then assessed the viability of HS683 and KNS89 cells after 24, 48, and 72 h of treatment with different concentrations of SHD. As a result, we found that SHD gradually decreased the cell viability of HS683 and KNS89 cells in a dose-dependent manner with increasing treatment time (Fig. 5A). Also, the colony formation assay was actualized to further explore the anti-proliferation role of SHD. The results suggested that the cloning ability of HS683 and KNS89 cells were remarkably inhibited with the increase of SHD (Fig. 5B). Moreover, treatment of 5, 10, 20 μg/mL of SHD significantly increased the apoptotic rates in HS683 and KNS89 cells, respectively, as shown in Fig. 5C. For the functions of SHD on cell proliferation and apoptosis on HS683 and KNS89 cells, the expression levels of apoptosis-related proteins Bax, Bcl-2, and Caspase-3 were verified by Western blot as shown in Fig. 5D (Fig. S1). Here, the results displayed that as the dose of SHD increased, the expression levels of Bax and Caspase-3 increased while Bcl-2 decreased. Meanwhile, the number of migrated and invaded HS683 and KNS89 cells was effectively decreased as the SHD dose increased in transwell assay (Fig. 6A–B). Considering that epithelial-to-mesenchymal transition (EMT) contributes to migration and invasion in cancer cells. Then, the expression levels of EMT-associated biomarkers E-cadherin and vimentin were measured using western blotting. The results showed that SHD increased the expression of E-cadherin and decreased the expression of vimentin in HS683 and KNS89 cells (Fig. 6C, Fig. S2). More importantly, the western blotting assay was performed to investigate the effects of SHD on protein expression levels of hub genes in HS683 and KNS89 cells. As exhibited in Fig. 6D (Fig. S3), the levels of PIK3CA, AKT1, CTNNB1, STAT3, EGFR, VEGFA, ERBB2, and HIF1A protein were decreased in HS683 and KNS89 cells, whereas TP53 had an elevated expression. In general, these results show that SHD might ameliorate the malignancy of glioma cells via the regulation of EMT and hub genes.

### 3.6 Effect of SHD on the tumor growth in mice

**Fig. 7:**
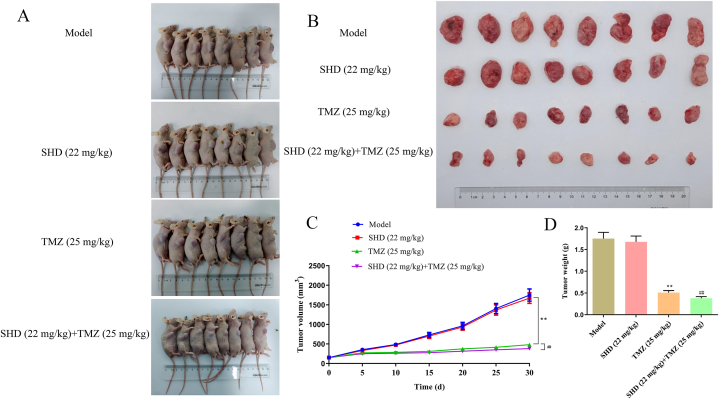
**SHD administration cooperated TMZ inhibited HS683 tumor growth in BALB/c-nu mice**. (A, B) BALB/c-nu mice (n = 8) with subcutaneous HS683-bearing tumors were intragastrically administrated with SHD (22 mg/kg), TMZ (25 mg/kg), and their combinations (SHD + TMZ). (C) Tumor volume was recorded every 5 days until 30 days. (D) After 30 days of treatment, the tumor tissues were collected and weighed. **P < 0.01 vs model group; ^##^*P < 0.01* vs TMZ (25 mg/kg) group.

**Fig. 8:**
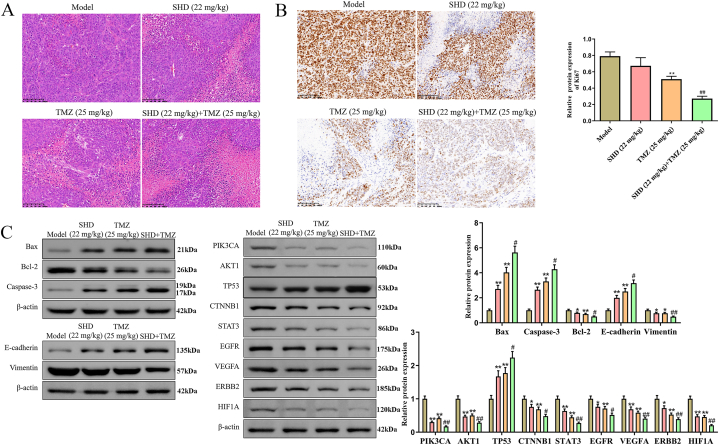
**SHD administration cooperated TMZ promoted glioma cell apoptosis in vivo**. (A) Representative pathological images of the xenograft tumors. Scale bar, 100 μm. (B) Immunohistochemistry and quantitative analysis of the expression of Ki67 in the xenograft tumor tissues (Scale bar = 100 μm). (C) Western blot and quantitative analysis of the protein expressions of EMT-related genes and overlapping genes in the tumor tissues. The unedited images are referenced in Fig. S4. ** = *P* < *0.05*, **P < *0.01* vs model group; ^##^*P* < *0.01* vs TMZ (25 mg/kg) group.

To investigate whether SHD has an antitumor effect in mice, HS683 tumor-bearing mice were treated with SHD or underwent peritoneal injection of TMZ, as well as their combination and the tumors were photographed and recorded, as shown in Fig. 7A–B. Moreover, TMZ treatment reduced tumor volume and weight compared to the model group; To everybody's surprise, there were no significant differences in SHD group with a slight reduction in this study (Fig. 7C–D). Interestingly, the results show that SHD might help in the inhibitory action of TMZ on the tumor volume and weight compared to the TMZ group. Also, the HE staining pointed out that SHD and TMZ treatment resulted in a large area of cell death in tumor tissues, especially the SHD + TMZ group (Fig. 8A). Meanwhile, SHD and TMZ treatment decreased the expression of Ki67 to a certain extent (Fig. 8B). And we also found that the Bcl2, vimentin, PIK3CA, AKT1, CTNNB1, STAT3, EGFR, VEGFA, ERBB2, and HIF1A expressions were suppressed in SHD, TMZ, and SHD + TMZ groups as well, while there was also an increase in the Bax, Caspase-3, E-cadherin, and TP53 in the treated groups (Fig. 8C, Fig. S4). Thus, these results suggested that SHD might be responsible for the enhancement of the anti-cancer effect of TMZ in glioma cells.

## 4 Discussion

Among brain tumors, glioma attracts much attention for its high incidence and malignancy. In recent decades, in addition to the standard treatment methods of surgical resection, radiotherapy combined with TMZ chemotherapy in the treatment of malignant glioma, and the intervention methods of genetics and immunology have achieved some research achievements. However, the overall treatment effect is not ideal. The reason is that malignant glioma grows in the way of infiltration into the surrounding normal tissues. Despite the continuous development and improved accuracy of neurosurgery, it is still difficult to completely remove the tumor tissue through surgery, which is easy to relapse, spread and invade after surgery, and eventually leads to the death of patients [32]. As one of the effective means of supplementary treatment of glioma, traditional Chinese medicine has played an effective role in the treatment of glioblastoma.

Although it has been confirmed that SHD has various effects on cell growth of glioma U87 and U251 cells through modern pharmacological studies [9]. Given its diverse active ingredients and complex mechanisms of action, multiple targets and signaling pathways need to be explored to gain insight into the therapeutic effects of the drug through regulatory biological networks. UPLC-Q-TOF/MS provides a large amount of fragment ion and elemental data to provide clues and a basis for the identification of unknown components of TCM. In particular, it should be emphasized that the method can analyze the components of complex systems comprehensively without splitting and neglecting any component, which is in line with the characteristics of the holistic and comprehensive effect of TCM.

In the current study, we used UPLC-Q-TOF/MS to characterize SHS and provide a basis for the material basis of the anti-migration and invasive effects of SHD on human glioma cells. Therefore, a total of 296 components were identified from SHD in positive ion mode and 165 components were identified from SHD in negative ion mode using UPLC-Q-TOF-MS/MS, most of which were flavonoids and terpenoids, including apigenin, daidzein, kaempferol, quercetin, biochanin A and so on. Among them, Shendge et al. reported that acacetin and apigenin as natural flavones triggered ROS-mediated apoptosis in U87 cells [33]. Similarly, Chen et al. illustrated that kaempferol inhibited cell proliferation by inducing autophagy and pyroptosis in U251 and U87 MG cells [34]. Generally, these findings suggested that the flavonoids contained in SHD are most likely the active components of SHD to inhibit proliferation, migration, and invasion and promote apoptosis of human malignant glioma cells.

Network pharmacology explores the relationship between drugs and diseases from a holistic perspective, which is consistent with the concept of Chinese medicine [35], and can enrich the experimental targets as much as possible from the perspective of data mining and help to elaborate the scientific connotation of the study. A total of 139 active components of SHD were identified via the network pharmacology analysis. After the combined screening of GenCliP 3, GeneCards, OMIM, and SHD component-predicted target genes, a total of 33 overlapping genes were found to be associated with pathway in cancer, proteoglycans in cancer, regulation of epithelial cell proliferation, gliogenesis, brain development, cell adhesion, autophagy, and cellular localization. Moreover, the AKT1, TP53, CTNNB1, STAT3, EGFR, VEGFA, PIK3CA, ERBB2, and HIF1A were identified as hub genes according to PPI network analysis. Also, the hub genes guide the drug components Dinatin, 6-Hydroxynaringenin, Rhamnazin, Eriodictyol, Luteolin, Naringenin, Diosgenin, Mairin, Farnesylacetone, Isorhamnetin, Diosgenin and so on. Most importantly, we found that the 9 hub genes were highly expressed in GBM and LGG, and the expression in LGG was positively correlated with poor prognosis. AKT1, a serine/threonine kinase, is a crucial component of the PI3K/Akt/mTOR pathway that enhances tumor formation, and its overexpression is linked to a bad outcome in GBM patients [36]. Gene mutations of a widely recognized tumor-suppressor gene, TP53, have been found in GBM [37], which reduces its anti-tumor effectiveness [38]. CTNNB1/β-catenin is a main effector of the canonical Wnt signaling, which plays a key role in the autophagy in GBM [39]. STAT3 is a critical transcription factor that sustained activation contributes to the maintenance of malignant characteristics in GBM [40]. EGFR, a transmembrane glycoprotein, is significantly overexpressed in GBM, which helps to promote cell survival, proliferation, and chemoresistance by activating the ERK1/2 and PI3K/Akt/mTOR pathways [41,42]. VEGFA with higher expression levels in the peritumoral brain zone is believed to be related to the recurrence of GBM [43]. Additionally, it is widely considered a central factor of pathological angiogenesis that is strongly linked to a bad prognosis in GBM patients [44]. PIK3CA is one of the homologous isoforms of Class IA PI3K that encode catalytic subunits p110α. Meanwhile, Brito et al. point out that PIK3CA mutations occur frequently and maintain cell survival in glioma recurrence [45]. ERBB2 belongs to the transmembrane protein receptor of the tyrosine kinase I subfamily and is highly expressed in various types of GBM [46,47]. HIF1A, a transcription factor, is activated in U251 and GL261 cells [48]. Also, Peng et al. found that HIF1A coordinates platelet-derived growth factor D and platelet-derived growth factor receptor subunit alpha expressions for activating AKT signaling to induce the malignancy of GBM [49]. Taken together, these findings might provide a deep mechanistic understanding and material basis of SHD against the development of glioma.

Besides, we performed the experimental verification on the effect of SHD *in vitro* or SHD + TMZ *in vivo* for fighting against GBM. In this study, we showed that SHD inhibited cell proliferation, migration, and invasion abilities, as well as promoted apoptosis of HS683 and KNS89 cells *in vitro.* Furthermore, when we combined SHD with TMZ to treat HS683 tumor-bearing mice, we found that the inhibition of tumor growth on SHD + TMZ was significantly strengthened compared to alone TMZ group, as accompanied by a reduction of Bcl-2, vimentin, PIK3CA, AKT1, CTNNB1, STAT3, EGFR, VEGFA, ERBB2, and HIF1A expressions and an increase of Bax, Caspase-3, E-cadherin, and TP53 levels *in vitro* and *in vivo*. At last, these results offer momentous initiatory evidence on the role of SHD in glioma treatment with a multiple-target pattern and suggest that SHD may be a promising candidate to raise TMZ sensitiveness for GMB treatment. However, the in-depth molecular mechanisms are far from fully clarified; more basic experiments ((e.g., mechanistic studies, clinical trials) and clinical translation (e.g., bioavailability, dosage optimization) are needed in our further research to enrich the scientific connotation of SHD.

## 5 Conclusion

In summary, we combined UPLC-Q-TOF/MS analysis, network pharmacology-based prediction, and verification experiments to identify the active compounds of SHD and to preliminarily inquire into the action targets of SHD. We demonstrated that SHD might inhibit the malignant behavior of GBM cells and promote TMZ sensitiveness to combat glioma by inactivating its targets. Altogether, our study showed that the anti-tumor mechanism of SHD to some extent relied on multi-component and multi-target patterns. Furthermore, further experiments are needed to develop more promising clinical benefits.

## Ethics statement

All experiments were accomplished in accordance with the guidelines of the Animal Care and Use Committee of Zhejiang Eyong Pharmaceutical Research and Development Center (Approve number: ZJEY-20220721-03).

## Funding statement

This work was supported by Shaoxing Municipal Science and Technology Plan Project of China (NO. 2022A14022) and Shaoxing Health Science and Technology Project of China (NO. 2022KY003).

## Data availability statement

The data used to support the findings of this study are available from the corresponding author upon request.

## CRediT authorship contribution statement

**Xiaobing Zhang:** Investigation, Conceptualization. **Xian Shao:** Investigation, Formal analysis. **Qingquan Bao:** Investigation. **Lingyan He:** Writing – review & editing, Investigation. **Xuchen Qi:** Supervision, Project administration, Funding acquisition, Conceptualization.

## Declaration of competing interest

The authors declare that they have no known competing financial interests or personal relationships that could have appeared to influence the work reported in this paper.
