# Supplementary material for: Integrated network pharmacology and experimental verification to reveal the role of Shezhi Huangling Decoction against glioma by inactivating PI3K/Akt-HIF1A axis
Source: Heliyon. 2024 Jul 6;10(14):e34215. doi: 10.1016/j.heliyon.2024.e34215 (PMC11292238; doi:10.1016/j.heliyon.2024.e34215)
Supplement: Multimedia component 1 [file mmc1.doc]

**Table S1 Identification of major chemical compounds in the aqueous extract of SHD under the positive ion mode**

| **NameEN** | **NameCN** | **Class** | **Formula** | **rtmed** |
| --- | --- | --- | --- | --- |
| 2,8-Dihydroxy-5,5,8-trimethyl-11-oxatetracyclo[7.3.1.0~1,9~.0~3,7~]tridecan-10-one |  | terpenoids(萜类) | C15H22O4 | 239.744 |
| 2',7-Dihydroxy-4'-methoxy-8-prenylflavan | 2'，7-二羟基-4'-甲氧基-8-丁烯基黄素 |  | C21H24O4 | 585.627 |
| 8-Hydroxy-7-methoxy-2H-1-benzopyran-2-one | 8-羟基-7-甲氧基-2H-1-苯并吡喃-2-酮 | phenylpropanoids(苯丙素类) | C10H8O4 | 953.7575 |
| 9-Hydroxycalabaxanthone | 氧杂蒽酮I标准品 |  | C24H24O6 | 397.23 |
| Adenine | 腺嘌呤 | alkaloid(生物碱) | C5H5N5 | 32.6286 |
| Anisic aldehyde | 对甲氧基苯甲醛 | Phenols(酚类) | C8H8O2 | 66.6331 |
| Apigenin | 芹菜素 | flavonoids(黄酮类) | C15H10O5 | 105.861 |
| Baicalein | 黄芩素 | flavonoids(黄酮类) | C15H10O5 | 267.304 |
| Benzoic acid | 苯甲酸 | Phenols(酚类) | C7H6O2 | 46.2958 |
| Carveol | 香芹醇;香芹酚 | Prenol lipids(异戊烯醇脂类) | C10H16O | 228.453 |
| Curcumenol | 莪术烯醇 | terpenoids(萜类) | C15H22O2 | 852.355 |
| Daidzein | 大豆苷元 | flavonoids(黄酮类) | C15H10O4 | 230.64 |
| Fallacinol | 迷人醇 | quinones(醌类) | C16H12O6 | 252.093 |
| Glyceryl linolenate | 亚麻酸甘油酯 |  | C21H36O4 | 648.43 |
| Goniothalenol | 哥纳香醇 | phenylpropanoids(苯丙素类) | C13H12O4 | 124.805 |
| Isoeugenol | 异丁子香酚 |  | C10H12O2 | 56.44105 |
| Kaempferol | 山柰酚 | flavonoids(黄酮类) | C15H10O6 | 166.521 |
| kojic acid | 曲酸 |  | C6H6O4 | 41.7283 |
| Lucidone C | 赤芝酮C | terpenoids(萜类) | C24H36O5 | 605.504 |
| Phenylacetaldehyde | 苯乙醛 | Phenols(酚类) | C8H8O | 679.063 |
| Prexanthoperol |  | terpenoids(萜类) | C20H26O3 | 528.28 |
| Quercetin | 槲皮素 | flavonoids(黄酮类) | C15H10O7 | 84.0721 |
| Rubiadin | 甲基异茜草素 | quinones(醌类) | C15H10O4 | 242.041 |
| Salicylic acid | 水杨酸 | Phenols(酚类) | C7H6O3 | 46.8193 |
| Scopoletin | 东莨菪内酯 | phenylpropanoids(苯丙素类) | C10H8O4 | 927.2815 |
| trans-Grandmarin | 反式香豆素 | phenylpropanoids(苯丙素类) | C15H16O6 | 327.384 |
| Wogonin | 汉黄芩素 | flavonoids(黄酮类) | C16H12O5 | 281.354 |
| Isoflavone base + 2O, 1MeO | 黄豆黄素 | flavonoids(黄酮类) | C16H12O5 | 267.304 |
| Biochanin A | 鹰嘴豆芽素A | flavonoids(黄酮类) | C16H12O5 | 248.598 |
| 7-Methoxy-4-methylcoumarin | 7-甲氧基-4-甲基香豆素 | phenylpropanoids(苯丙素类) | C11H10O3 | 163.77 |
| Formononetine | 芒柄花黄素 | flavonoids(黄酮类) | C16H12O4 | 305.881 |
| Tectochrysin | 杨芽黄素 | flavonoids(黄酮类) | C16H12O4 | 187.812 |
| Isofraxidin | 异嗪皮啶 | phenylpropanoids(苯丙素类) | C11H10O5 | 111.505 |
| 2-Methylbenzaldehyde | 2-甲基苯甲醛 |  | C8H8O | 397.23 |
| 7-hydroxy-1,4a-dimethyl-9-oxo-7-propan-2-yl-2,3,4,4b,5,6,10,10a-octahydrophenanthrene-1-carboxylic acid |  | terpenoids(萜类) | C20H30O4 | 397.783 |
| Erinacine C | 猴头菌多醇C | terpenoids(萜类) | C25H38O6 | 562.711 |
| Betaine | 甜菜碱 | alkaloid(生物碱) | C5H11NO2 | 954.324 |
| Kaempferide | 山奈素 | flavonoids(黄酮类) | C16H12O6 | 145.2785 |
| 8-hydroxy-5,7-dimethoxy-2-phenyl-2,3-dihydrochromen-4-one |  | flavonoids(黄酮类) | C17H16O5 | 318.06 |
| 8-Acetoxy-4-acoren-3-one | 8-乙酰氧基-4-乙酰基-3-酮 | terpenoids(萜类) | C17H26O3 | 273.069 |
| Nicotinic acid | 烟酸 | alkaloid(生物碱) | C6H5NO2 | 366.112 |
| Emodin | 大黄素 | quinones(醌类) | C15H10O5 | 159.5 |
| Coumaric acid |  | phenylpropanoids(苯丙素类) | C9H8O3 | 50.9376 |
| Proline | 脯氨酸 | alkaloid(生物碱) | C5H9NO2 | 32.9658 |
| 24-Hydroxyglycyrrhetic acid | 24-羟基甘草次酸 | terpenoids(萜类) | C30H46O5 | 325.072 |
| Isorhamnetin | 异鼠李素 | flavonoids(黄酮类) | C16H12O7 | 208.9695 |
| Phenylalanine | 苯丙氨酸 | Amino acid derivatives(氨基酸衍生物) | C9H11NO2 | 54.4895 |
| 5-hydroxy-2,2-dimethyl-10-(2-methylbut-3-en-2-yl)pyrano[3,2-g]chromen-8-one |  | phenylpropanoids(苯丙素类) | C19H20O4 | 468.405 |
| Flavone base + 2O, 1MeO, C-Hex |  | flavonoids(黄酮类) | C22H22O10 | 261.339 |
| 2,3-Secoporrigenin | 2,3-二氯氰菊酯 | terpenoids(萜类) | C27H40O6 | 700.427 |
| N-(14-Methylhexadecanoyl)pyrrolidine | N-(14甲基十六烷基)吡咯烷 |  | C21H41NO | 833.643 |
| Valine | 缬氨酸 | alkaloid(生物碱) | C5H11NO2 | 597.648 |
| Methyl 2-furoate | 2-糠酸甲酯 |  | C6H6O3 | 914.905 |
| Herbacetin | 草质素 | flavonoids(黄酮类) | C15H10O7 | 128.905 |
| 2'-Hydroxyacetophenone | 2’-羟基苯乙酮 |  | C8H8O2 | 90.9815 |
| trans-4-Coumaric acid |  | phenylpropanoids(苯丙素类) | C9H8O3 | 82.9299 |
| Maltol | 麦芽酚 | flavonoids(黄酮类) | C6H6O3 | 954.984 |
| Prunetin | 樱黄素 | flavonoids(黄酮类) | C16H12O5 | 304.766 |
| Isoliquiritigenin | 异甘草素 | flavonoids(黄酮类) | C15H12O4 | 117.003 |
| 6beta-Hydroxy-3-oxo-12-oleanen-28-oic acid | 6Beta-羟基-3-氧代-12-油酸-28-油酸 | terpenoids(萜类) | C30H46O4 | 598.26 |
| Ginsenoyne E | 人参炔E |  | C17H22O2 | 865.344 |
| Naringenin | 柚皮素 | flavonoids(黄酮类) | C15H12O5 | 142.081 |
| Choline chloride | L-胆碱 |  | C5H14NO | 50.2966 |
| Atractylenolide III | 白术内酯Ⅲ | terpenoids(萜类) | C15H20O3 | 407.058 |
| Dipropylphthalate | 邻苯二甲酸二丙酯 |  | C14H18O4 | 250.858 |
| Lucidenic acid M | 赤芝酸M | terpenoids(萜类) | C27H42O6 | 663.579 |
| cuminyl alcohol | 4-异丙基苯甲醇 | Prenol lipids(异戊烯醇脂类) | C10H14O | 156.269 |
| Aurantio-obtusin | 橙黄决明素 | quinones(醌类) | C17H14O7 | 274.234 |
| 9-Acetoxyfukinanolide | 9-乙酰氧基蜂斗菜次螺内酯 | terpenoids(萜类) | C17H24O4 | 263.088 |
| Talatisamine | 塔拉萨敏 | terpenoids(萜类);alkaloid(生物碱) | C24H39NO5 | 651.019 |
| (E/Z)-cinnamic acid | 肉桂酸 | phenylpropanoids(苯丙素类) | C9H8O2 | 137.805 |
| Allyl benzoate | 苯甲酸烯丙酯 |  | C10H10O2 | 573.488 |
| Reichsteins substance S | 孕甾-4-烯-17Alpha,21-二醇-3,20-二酮-21-醋酸酯 | terpenoids(萜类) | C21H30O4 | 566.444 |
| (4S,5Z,6S)-4-(2-methoxy-2-oxoethyl)-5-[2-[(E)-3-phenylprop-2-enoyl]oxyethylidene]-6-[(2S,3R,4S,5S,6R)-3,4,5-trihydroxy-6-(hydroxymethyl)oxan-2-yl]oxy-4H-pyran-3-carboxylic acid |  | terpenoids(萜类) | C15H20O3 | 291.308 |
| 3-Hydroxyflavone | 3-羟基黄酮 | flavonoids(黄酮类) | C15H10O3 | 374.618 |
| 2-Phenylethanol | 苯乙醇 |  | C8H10O | 41.4723 |
| Sissotrine |  | flavonoids(黄酮类) | C22H22O10 | 225.535 |
| Naringenin chalcone | 柚皮苷查尔酮 | flavonoids(黄酮类) | C15H12O5 | 86.15975 |
| Arginine | 精氨酸 | Amino acid derivatives(氨基酸衍生物) | C6H14N4O2 | 32.9658 |
| 2-Propylphenol | 2-丙基苯酚 |  | C9H12O | 144.621 |
| Hordenine | 大麦芽碱 | alkaloid(生物碱) | C10H15NO | 15.3025 |
| m-Xylene |  |  | C8H10 | 469.533 |
| Abscisic acid | 脱落酸 | terpenoids(萜类) | C15H20O4 | 208.348 |
| Isoleucine | 异亮氨酸 | Amino acid derivatives(氨基酸衍生物) | C6H13NO2 | 63.8244 |
| Vanillic acid | 香草酸 | Organic acids and derivatives(有机酸及其衍生物) | C8H8O4 | 25.1754 |
| Dihydrokaempferol | 二氢山柰酚 | flavonoids(黄酮类) | C15H12O6 | 154.692 |
| Flavanone base + 3O, 1Prenyl |  | flavonoids(黄酮类) | C20H20O5 | 303.042 |
| Acetic anhydride | 醋酸酐 |  | C4H6O3 | 164.308 |
| Icaritin | 淫羊藿素 | flavonoids(黄酮类) | C21H20O6 | 420.334 |
| p-Hydroxybenzaldehyde | 对羟基安息香醛 | Phenols(酚类) | C7H6O2 | 76.8565 |
| p-Mentha-1,3,8-triene | p-薄荷-1,3,8-三烯 |  | C10H14 | 163.193 |
| 12-Ketoporrigenin | 12-酮孔菌素 | terpenoids(萜类);steroidal saponin(甾体皂苷类) | C27H42O5 | 717.8955 |
| Cyclo(leucylprolyl) |  |  | C11H18N2O2 | 97.217 |
| Chrysin | 白杨素 | flavonoids(黄酮类) | C15H10O4 | 90.0047 |
| Vanillin | 香兰素 | Phenols(酚类) | C8H8O3 | 92.6306 |
| Caffeic acid | 咖啡酸 | phenylpropanoids(苯丙素类) | C9H8O4 | 52.8342 |
| Ononin | 芒柄花苷 | flavonoids(黄酮类) | C22H22O9 | 206.209 |
| Trilobatin | 三叶苷 | flavonoids(黄酮类) | C21H24O10 | 122.041 |
| Beta-Caryophyllene Alcohol |  | terpenoids(萜类) | C15H26O | 741.177 |
| Cichoralexin | 环磷酰胺 | terpenoids(萜类) | C15H20O3 | 326.804 |
| 8-Methoxypsoralen | 花椒毒素 | phenylpropanoids(苯丙素类) | C12H8O4 | 93.1304 |
| Coumaperine | 香豆素 | alkaloid(生物碱) | C16H19NO2 | 888.134 |
| 3beta,6beta-Dihydroxynortropane | 菪烷 | alkaloid(生物碱) | C7H13NO2 | 32.6286 |
| Ginkgolide A |  | terpenoids(萜类) | C20H24O9 | 231.06 |
| 2',4'-Dihydroxychalcone |  | flavonoids(黄酮类) | C15H12O3 | 958.077 |
| Ethylbenzene |  |  | C8H10 | 426.929 |
| Sophoricoside | 槐角苷 | flavonoids(黄酮类) | C21H20O10 | 107.87 |
| Flavanone base +2O, 1MeO | 樱花亭 | flavonoids(黄酮类) | C16H14O5 | 184.473 |
| 3,4-Dimethoxybenzaldehyde |  | Aromaticity(芳香族化合物) | C9H10O3 | 51.3841 |
| Phthalic anhydride | 苯酐 |  | C8H4O3 | 57.1894 |
| Withaphysacarpin |  | terpenoids(萜类) | C28H40O7 | 284.788 |
| 3-Formylindole | 3-甲酰基吲哚 | alkaloid(生物碱) | C9H7NO | 132.016 |
| 1,4,5-Naphthalenetriol | 1,4,5-萘三醇 |  | C10H8O3 | 257.84 |
| Pyroglutamic acid (not validated, isomer of 88) | 焦谷氨酸 | Amino acid derivatives(氨基酸衍生物) | C5H7NO3 | 40.9883 |
| 2-Phenylethyl beta-D-glucopyranoside | 2-苯乙基Beta-D-葡萄糖苷 |  | C14H20O6 | 137.805 |
| (+)-Fargesin | 辛夷脂素 | phenylpropanoids(苯丙素类) | C21H22O6 | 151.451 |
| Genistein | 染料木素 | flavonoids(黄酮类) | C15H10O5 | 232.786 |
| Umbelliferone | 伞形花内酯 | phenylpropanoids(苯丙素类) | C9H6O3 | 239.744 |
| Pterosin O | 含蕨素 O |  | C15H20O2 | 304.766 |
| (+/-)-Jasmonic acid | 茉莉酸 | Fatty acids(脂肪酸类) | C12H18O3 | 250.276 |
| Loliolide | 地芰普内酯 | terpenoids(萜类) | C11H16O3 | 101.398 |
| 2-(3,4-dihydroxyphenyl)-5,7-dihydroxy-2,3-dihydrochromen-4-one |  | flavonoids(黄酮类) | C15H12O6 | 112.01 |
| Santene | 檀烯 |  | C9H14 | 339.225 |
| Geranial | (E)-3,7-二甲基-2,6-辛二烯醛 |  | C10H16O | 246.407 |
| Germacrone | 吉马酮 | terpenoids(萜类) | C15H22O | 584.369 |
| alpha-Linolenic acid | -亚麻酸 | Aliphatic acyl(脂肪酰类) | C18H30O2 | 475.067 |
| 2-Hydroxyadenine | 2-羟基腺嘌呤 | alkaloid(生物碱) | C5H5N5O | 32.6286 |
| Khellin | 呋喃并色酮 | flavonoids(黄酮类) | C14H12O5 | 293.629 |
| (R)-Campholenic aldehyde | 龙脑烯醛 |  | C10H16O | 29.2707 |
| Resveratrol | 白藜芦醇 | Phenols(酚类) | C14H12O3 | 161.476 |
| Daidzein-8-C-glucoside |  | flavonoids(黄酮类) | C21H20O9 | 44.7173 |
| 1,5,9-trihydroxy-5,7,7-trimethyl-4,5a,6,8,8a,9-hexahydro-1H-azuleno[5,6-c]furan-3-one |  | terpenoids(萜类) | C15H22O5 | 363.724 |
| 2-Hydroxy-4-methoxybenzaldehyde | 4-甲氧基水杨醛 | Phenols(酚类) | C8H8O3 | 935.8965 |
| Licoricesaponin H2 |  | terpenoids(萜类) | C42H62O16 | 350.588 |
| 2-Hexyl-5-[2-(4-hydroxy-3-methoxyphenyl)ethyl]furan | 2-己基-5-[2-(4-羟基-3-甲氧基苯基)乙基]呋喃 |  | C19H26O3 | 549.931 |
| Panaquinquecol 1 |  |  | C18H28O3 | 256.677 |
| Ethyl 4-methoxycinnamate | 对甲氧基肉桂酸乙酯 | phenylpropanoids(苯丙素类) | C12H14O3 | 40.5734 |
| 3-Methylellagic acid 8-rhamnoside | 关键词：3-甲基丙酸8-大麦糖 | phenylpropanoids(苯丙素类) | C21H18O12 | 170.924 |
| Fisetin | 漆黄素 | flavonoids(黄酮类) | C15H10O6 | 103.34255 |
| Ganoderiol F | 紫苏A | terpenoids(萜类) | C30H46O3 | 328.557 |
| alpha-Hydrojuglone 4-O-b-D-glucoside | Alpha-水胡桃醌4-O-b-D-葡萄糖苷 |  | C16H18O8 | 200.28 |
| Thermophillin |  | quinones(醌类) | C8H8O4 | 947.001 |
| (E)-3-(2-Hydroxyphenyl)-2-propenal | 3-(2-羟基苯基)-2-丙烯醛 |  | C9H8O2 | 312.294 |
| Phenethylacetate | 乙酸苯乙酯 | Aromaticity(芳香族化合物) | C10H12O2 | 177.479 |
| Wedelolactone | 蟛蜞菊内酯 | phenylpropanoids(苯丙素类) | C16H10O7 | 135.082 |
| 5,7-dihydroxy-2-(4-hydroxyphenyl)-6-[3,4,5-trihydroxy-6-(hydroxymethyl)oxan-2-yl]-4H-chromen-4-one |  | flavonoids(黄酮类) | C21H20O10 | 38.0106 |
| Scoparone | 滨蒿內酯 | phenylpropanoids(苯丙素类) | C11H10O4 | 86.3369 |
| Licochalcone A | 甘草查尔酮A | flavonoids(黄酮类) | C21H22O4 | 439.319 |
| (+)-Alantolactone | 土木香内酯 | terpenoids(萜类) | C15H20O2 | 484.4835 |
| Maslinic acid | 山楂酸 | terpenoids(萜类) | C30H48O4 | 426.929 |
| 2-Cyclohexen-1-one, 4-hydroxy-4-(3-hydroxybutyl)-3,5,5-trimethyl- | 2-环己烯-1-酮，4-羟基-4-（3-羟基丁基）-3,5,5-三甲基- |  | C13H22O3 | 233.363 |
| Azuleno[5,6-c]furan-1(3H)-one, 4,4a,5,6,7,7a,8,9-octahydro-4,8-dihydroxy-6,6,8-trimethyl- |  | terpenoids(萜类) | C15H22O4 | 357.696 |
| N-cis-Feruloyltyramine | N-顺-阿魏酰酪胺 |  | C18H19NO4 | 189.435 |
| Polyporusterone B | 多孔菌甾酮B | terpenoids(萜类) | C28H44O6 | 696.268 |
| isoimperatorin | 异欧前胡素 | phenylpropanoids(苯丙素类) | C16H14O4 | 183.506 |
| Periplocymarin | 杠柳次苷 | terpenoids(萜类);cardiac glycosides(强心苷类) | C30H46O8 | 664.758 |
| 1beta-Hydroxyalantolactone | 1Beta-羟基丙氨酸内酯 | terpenoids(萜类) | C15H20O3 | 278.092 |
| Isoferulic acid | 异阿魏酸 | phenylpropanoids(苯丙素类) | C10H10O4 | 156.839 |
| Spirostane -2H, + 1O, O-Hex-dHex, C6H9O4 |  | terpenoids(萜类) | C45H70O17 | 772.57 |
| 3-(2-Hydroxy-3,4-dimethoxyphenyl)-7-chromanol |  | flavonoids(黄酮类) | C17H18O5 | 331.609 |
| Zedoarol |  | terpenoids(萜类) | C15H18O3 | 279.036 |
| Coniferyl aldehyde | 松柏醛 | phenylpropanoids(苯丙素类) | C10H10O3 | 78.5744 |
| 7-Galloylcatechin | 7-没食子酰基儿茶素 |  | C22H18O10 | 92.0611 |
| 3-propan-2-yl-2,3,6,7,8,8a-hexahydropyrrolo[1,2-a]pyrazine-1,4-dione | 环(脯氨酸-缬氨酸)二肽 |  | C10H16N2O2 | 51.3841 |
| Furanofukinin | 呋喃诺福金 | terpenoids(萜类) | C16H24O2 | 546.789 |
| (4Z,7Z)-5,9,9-Trimethyl-11-oxabicyclo[8.2.1]trideca-1(13),4,7-triene-6,12-dione | （4Z，7Z）-5,9,9-三甲基-11-氧杂环[8.2.1]十三碳-1（13），4,7-三烯-6,12-二酮 |  | C15H18O3 | 204.966 |
| beta-Elemonic acid | 岚香酮酸 | terpenoids(萜类) | C30H46O3 | 405.9 |
| Dimethyl succinate | 丁二酸二甲酯 |  | C6H10O4 | 103.07305 |
| Lysionotin | 石吊兰素 | flavonoids(黄酮类) | C18H16O7 | 335.691 |
| Liquiritin | 甘草苷 | flavonoids(黄酮类) | C21H22O9 | 173.617 |
| Propranolol |  | Aromaticity(芳香族化合物) | C16H21NO2 | 899.404 |
| Gingerol | 姜辣素 |  | C17H26O4 | 384.821 |
| trans-pterostilbene | 顺式紫檀芪 | Phenols(酚类) | C16H16O3 | 448.445 |
| Methylparaben | 尼泊金甲 | Phenols(酚类) | C8H8O3 | 921.031 |
| Guan-fu base Y | 关附辛素 | terpenoids(萜类);alkaloid(生物碱) | C22H29NO5 | 597.648 |
| P-Anisic acid | 对甲氧基苯甲酸 | Phenols(酚类) | C8H8O3 | 55.4687 |
| Ganoderic acid DM | 灵芝酸DM | terpenoids(萜类) | C30H44O4 | 248.598 |
| Zizyberanalic acid |  | terpenoids(萜类) | C30H46O4 | 456.038 |
| Phloretin | 根皮素 | flavonoids(黄酮类) | C15H14O5 | 399.375 |
| Isolicoflavonol |  | flavonoids(黄酮类) | C20H18O6 | 392.025 |
| Dihydroactinidiolide | 双氢猕猴桃交酯 |  | C11H16O2 | 302.457 |
| 5-Hydroxymethylfurfural | 5-羟甲基糠醛 |  | C6H6O3 | 43.3669 |
| Cearoin |  | Phenols(酚类) | C14H12O4 | 246.936 |
| Procurcumadiol | 原莪术二醇 | terpenoids(萜类) | C15H22O3 | 281.933 |
| 4-Hydroxybenzoylcholine | 4-羟基苯甲酰胆碱 | alkaloid(生物碱) | C12H18NO3 | 929.739 |
| Curzerenone | 莪术酮 |  | C15H18O2 | 457.904 |
| 4-Butyl-gamma-butyrolactone | 丙位辛内酯 |  | C8H14O2 | 426.929 |
| Casticin | 蔓荆子黄素 | flavonoids(黄酮类) | C19H18O8 | 333.386 |
| 3,19-Dihydroxyurs-12-ene-23,28-dioic acid |  | terpenoids(萜类) | C30H46O6 | 774.886 |
| Licoricesaponin G2 | 甘草皂苷G3 | terpenoids(萜类) | C42H62O17 | 369.722 |
| Ethylparaben |  | Phenols(酚类) | C9H10O3 | 318.06 |
| Isophorone | 异佛尔酮 |  | C9H14O | 165.405 |
| 6-methoxy-7-(3-methylbut-2-enoxy)chromen-2-one | 6-甲氧基-7-异戊烯氧基香豆素 | phenylpropanoids(苯丙素类) | C15H16O4 | 360.639 |
| Benzenepropanamide, N-[2-(acetyloxy)-1-(phenylmethyl)ethyl]-alpha-(benzoylamino)- |  |  | C27H28N2O4 | 441.878 |
| 3,8a-Dihydroxy-5-isopropylidene-3,8-dimethyl-2,3,3a,4,5,8a-hexahydro-6(1H)-azulenone |  | terpenoids(萜类) | C15H22O3 | 292.474 |
| Dihydro-Quer | 二氢槲皮素 | flavonoids(黄酮类) | C15H12O7 | 103.07305 |
| Corosolic acid | 科罗索酸 | terpenoids(萜类) | C30H48O4 | 348.588 |
| Pyridoxine |  | alkaloid(生物碱) | C8H11NO3 | 36.8523 |
| DL-Pipecolinic acid |  | alkaloid(生物碱) | C6H11NO2 | 32.9658 |
| Isovanillic acid | 异香草酸 | Phenols(酚类) | C8H8O4 | 62.1754 |
| Isospathulenol |  | terpenoids(萜类) | C15H24O | 369.118 |
| Licoricesaponin E2 | 甘草皂甙E2 | terpenoids(萜类) | C42H60O16 | 322.838 |
| Levistilide A | 欧当归内酯A | terpenoids(萜类) | C24H28O4 | 597.648 |
| 6-[3-[(3,4-dimethoxyphenyl)methyl]-4-methoxy-2-(methoxymethyl)butyl]-4-methoxy-1,3-benzodioxole |  | phenylpropanoids(苯丙素类) | C24H32O7 | 488.789 |
| Flavanone base + 6O |  | flavonoids(黄酮类) | C15H12O8 | 167.092 |
| 3 Hydroxycoumarin | 3-羟基香豆素 |  | C9H6O3 | 45.3841 |
| Sojagol |  | phenylpropanoids(苯丙素类) | C20H16O5 | 525.097 |
| Histamine |  | alkaloid(生物碱) | C5H9N3 | 32.6286 |
| Pantothenic acid (not validated) | 泛酸 |  | C9H17NO5 | 35.9773 |
| Cafestol | 咖啡醇 | terpenoids(萜类) | C20H28O3 | 386.71 |
| Methoxyeugenol | 4-烯丙基-2,6-二甲氧基苯酚 |  | C11H14O3 | 185.541 |
| Panaquinquecol 2 |  |  | C17H24O3 | 575.686 |
| 4-Ethyl-2-methoxyphenol | 4-乙基-2-甲氧基苯酚 |  | C9H12O2 | 68.3058 |
| TYROSINE | 酪氨酸 | Amino acid derivatives(氨基酸衍生物) | C9H11NO3 | 32.6286 |
| Dibutylphthalate | 邻苯二甲酸二丁酯 | Organic acids and derivatives(有机酸及其衍生物) | C16H22O4 | 603.149 |
| Lucidenic acid C | 赤芝酸C | terpenoids(萜类) | C27H40O7 | 341.696 |
| Sinapoylhexoside | 芥子基己糖苷 | phenylpropanoids(苯丙素类) | C17H22O10 | 62.7355 |
| blennin C | 3-[2-(4,4-二甲基-1-环戊烯-1-基)丙基]-4-(羟基甲基)呋喃-2(5h)-酮 | terpenoids(萜类) | C15H22O3 | 161.476 |
| 3,5-Dimethoxy-4-hydroxybenzaldehyde | 丁香醛 | Phenols(酚类) | C9H10O4 | 99.8236 |
| Schizandrin | 五味子素C(五味子丙素) | phenylpropanoids(苯丙素类) | C24H32O7 | 447.223 |
| Pesticide3_Propoxur_C11H15NO3_Baygon |  |  | C11H15NO3 | 44.7173 |
| Dihydrovaltrate | 地戊曲酯 |  | C22H32O8 | 810.471 |
| Glabrone | 光甘草酮 | flavonoids(黄酮类) | C20H16O5 | 509.146 |
| Rubrofusarin | 柔红霉素 |  | C15H12O5 | 201.53 |
| 7-Hydroxy-1,7-bis(4-hydroxy-3-methoxyphenyl)-1-heptene-3,5-dione | 7-羟基-1,7-双(4-羟基-3-甲氧基苯基)-1-庚-3,5-二酮 |  | C21H22O7 | 259.591 |
| (S)-Bilobanone | (S)-银杏酮 |  | C15H20O2 | 176.442 |
| Paraldehyde | 三聚乙醛 |  | C6H12O3 | 648.903 |
| Epoxyganoderiol A | 环氧灵芝醇A | terpenoids(萜类) | C30H48O4 | 445.438 |
| (1alpha,6alpha,7alphaH)-2,4(15)-Copadiene | (1alpha，6alpha，7alphaH)-2,4(15)-连二烯 | terpenoids(萜类) | C15H22 | 265.161 |
| Kaempferol-3-O-glucuronoside |  | flavonoids(黄酮类) | C21H18O12 | 203.841 |
| Grandisin | 大楼子素 | phenylpropanoids(苯丙素类) | C24H32O7 | 460.953 |
| Kaurane-17,18-dioic acid |  | terpenoids(萜类) | C20H30O4 | 318.06 |
| Rosmadial | 迷迭香双醛 | terpenoids(萜类) | C20H24O5 | 327.384 |
| Methylisoeugenol |  |  | C11H14O2 | 1.60559 |
| 1alpha-Hydroxyarbusculin A | 1Alpha-羟基脲醛 | terpenoids(萜类) | C15H22O4 | 240.893 |
| 4-Nitrophenol | 4-硝基苯酚 | Phenols(酚类) | C6H5NO3 | 33.8404 |
| Mesaconitine | 中乌头碱 | alkaloid(生物碱);terpenoids(萜类) | C33H45NO11 | 308.195 |
| Semilicoisoflavone B |  | flavonoids(黄酮类) | C20H16O6 | 376.373 |
| Matricin | 母菊素 | terpenoids(萜类) | C17H22O5 | 178.609 |
| Isobornyl propionate | 丙酸异龙脑酯 | terpenoids(萜类) | C13H22O2 | 107.331 |
| 3'-Hydroxy-4'-methoxyglabridin |  |  | C21H22O5 | 455.418 |
| Deacetylnomilin | 去乙酰闹米林 | terpenoids(萜类) | C26H32O8 | 436.22 |
| Dihydrocapsaicin | 二氢辣椒碱 | alkaloid(生物碱) | C18H29NO3 | 240.321 |
| Ceanothic acid | 美洲茶酸 | terpenoids(萜类) | C30H46O5 | 482.113 |
| 4,4,8,10,14-pentamethyl-17-(4,5,6-trihydroxy-6-methylheptan-2-yl)-2,5,6,7,9,15-hexahydro-1H-cyclopenta[a]phenanthrene-3,16-dione |  | terpenoids(萜类) | C30H46O5 | 339.5865 |
| Neobavaisoflavone | 新补骨脂异黄酮 | flavonoids(黄酮类) | C20H18O4 | 392.614 |
| 2-Naphthaleneacetic acid, decahydro-8-hydroxy-4a,8-dimethyl-alpha-methylene- |  | terpenoids(萜类) | C15H24O3 | 817.294 |
| Epinepetalactone | 反式-顺式-荆芥内酯 |  | C10H14O2 | 45.6126 |
| Uralsaponin B | 乌拉尔甘草皂苷乙 | terpenoids(萜类) | C42H62O16 | 443.8825 |
| Auxin b | 茁长素b | terpenoids(萜类) | C18H30O4 | 311.9645 |
| DL-Coniine |  | alkaloid(生物碱) | C8H17N | 898.326 |
| T-2 Toxin |  | terpenoids(萜类) | C24H34O9 | 245.736 |
| Flavone base + 3O, C-Hex-dHex |  | flavonoids(黄酮类) | C27H30O14 | 44.24245 |
| Pteroside A | 含蕨素 A |  | C21H30O8 | 125.377 |
| 2,3-Pentanedione | 2,3-戊二酮 |  | C5H8O2 | 12.9566 |
| Flavone base + 3O, O-HexA |  | flavonoids(黄酮类) | C21H18O11 | 167.347 |
| Chrysin Dimethyl Ether |  | flavonoids(黄酮类) | C17H14O4 | 438.076 |
| 5'-(furan-3-yl)-4a-hydroxy-4,7-dimethylspiro[5,6,7,8a-tetrahydro-1H-naphthalene-8,3'-oxolane]-2,2'-dione |  | terpenoids(萜类) | C19H22O5 | 108.998 |
| Cinnamaldehyde | 桂皮醛 | phenylpropanoids(苯丙素类) | C9H8O | 33.346 |
| (-)-12-Hydroxyjasmonic acid | 12-羟基茉莉酸 | Jasmonic acid(茉莉酸类) | C12H18O4 | 80.2235 |
| Ganoderol A | 灵芝醇A | terpenoids(萜类) | C30H46O2 | 319.816 |
| Verbascoside | 毛蕊花糖苷 | phenylpropanoids(苯丙素类) | C29H36O15 | 128.905 |
| 1-Naphthalenecarboxylic acid, 5-[2-(2,5-dihydro-2-oxo-3-furanyl)ethyl]decahydro-1,4a-dimethyl-6-methylene- |  | terpenoids(萜类) | C20H28O4 | 417.896 |
| L-2,3-DIAMINOPROPIONIC ACID | L-2,3-二氨基丙酸 |  | C3H8N2O2 | 1.60559 |
| Isosativan | (R)-2-(3,4-二氢-7-甲氧基-2H-1-苯并吡喃-3-基)-5-甲氧基苯酚 |  | C17H18O4 | 294.127 |
| 8-Hydroxy-2-methoxy-6-methyl-1,4-naphthoquinone | 8-羟基-2-甲氧基-6-甲基-1,4-萘醌 | quinones(醌类) | C12H10O4 | 170.924 |
| Licoricone | 甘草利酮 | flavonoids(黄酮类) | C22H22O6 | 504.186 |
| 5-hydroxy-2-(4-hydroxyphenyl)-7-(3,4,5-trihydroxy-6-methyloxan-2-yl)oxy-3-(3,4,5-trihydroxyoxan-2-yl)oxychromen-4-one |  | flavonoids(黄酮类) | C26H28O14 | 69.4541 |
| Methyl vanillate | 香草酸甲酯 | Phenols(酚类) | C9H10O4 | 913.735 |
| 2,3-dihydroxypropyl hexadecanoate | 甘油单棕榈酸酯 |  | C19H38O4 | 749.997 |
| Oleanane -4H, + 2O | 大豆皂醇 C | terpenoids(萜类) | C30H48O2 | 393.199 |
| cis-Miyabenol C |  |  | C42H32O9 | 233.363 |
| Asiatic Acid |  | terpenoids(萜类) | C30H48O5 | 283.67 |
| Cadabicilone |  | terpenoids(萜类) | C15H22O3 | 458.462 |
| Isomyristicin | 异肉豆蔻碱 |  | C11H12O3 | 88.8617 |
| Saussurea lactone | 雪莲内酯 | terpenoids(萜类) | C15H22O2 | 294.127 |
| (6beta,8alpha)-6-Hydroxy-7(11)-eremophilen-12,8-olide |  | terpenoids(萜类) | C15H22O3 | 175.895 |
| gamma-Eudesmol rhamnoside |  | terpenoids(萜类) | C21H36O5 | 292.474 |
| Isotrifoliol | 异利福醇 | phenylpropanoids(苯丙素类) | C16H10O6 | 190.582 |
| 2,3-Dehydrosilybin | 2,3-脱氢水飞蓟宾 | flavonoids(黄酮类) | C25H20O10 | 323.893 |
| Hydroxytanshinone | 羟基丹参酮IIA |  | C19H18O4 | 519.268 |
| Dihydroisoalantolactone | 二氢异土木香内酯 | terpenoids(萜类) | C15H22O2 | 333.386 |
| Cuelure | 乙酸覆盆子酮酯 |  | C12H14O3 | 181.844 |
| 7-Hydroxyterpineol 8-glucoside | 7-羟基松油醇8-葡萄糖苷 |  | C16H28O7 | 152.008 |
| Soybean saponin fraction B1 | 大豆皂苷 | terpenoids(萜类) | C48H78O18 | 394.371 |
| Isorhamnetin 3-robinobioside | 异鼠李素-3-O-刺槐二糖苷 | flavonoids(黄酮类) | C28H32O16 | 175.895 |
| Kanzonol F |  |  | C26H28O5 | 524.461 |
| Licoricesaponin K2 | 甘草皂甙K2 | terpenoids(萜类) | C42H62O16 | 457.904 |
| 1'-Acetoxychavicol acetate | 1'-乙酰氧基胡椒酚乙酸酯 |  | C13H14O4 | 249.133 |
| Isoacteoside | 异麦角甾苷 | phenylpropanoids(苯丙素类) | C29H36O15 | 128.905 |
| Armexifolin | 阿米西林 | terpenoids(萜类) | C15H18O4 | 104.085 |
| Neoisoliquiritin | 新异甘草苷 | flavonoids(黄酮类) | C21H22O9 | 116.429 |
| 3-Hydroxy-5-isopropylidene-3,8-dimethyl-2,3,3a,4,5,8a-hexahydro-6(1H)-azulenone | 原莪术烯醇 | terpenoids(萜类) | C15H22O2 | 357.108 |
| Kanzonol K |  | flavonoids(黄酮类) | C26H28O6 | 460.953 |
| Citrusinol | 柠檬酚 | flavonoids(黄酮类) | C20H16O6 | 398.305 |
| Diisobutyl phthalate | 邻苯二甲酸二异丁酯 |  | C16H22O4 | 367.912 |
