# Supplementary material for: Integrated network pharmacology and experimental verification to reveal the role of Shezhi Huangling Decoction against glioma by inactivating PI3K/Akt-HIF1A axis
Source: Heliyon. 2024 Jul 6;10(14):e34215. doi: 10.1016/j.heliyon.2024.e34215 (PMC11292238; doi:10.1016/j.heliyon.2024.e34215)
Supplement: Multimedia component 2 [file mmc2.doc]

**Table S2 Identification of major chemical compounds in the aqueous extract of SHD under the negative ion mode**

| **NameEN** | **NameCN** | **Class** | **Formula** | **rtmed** |
| --- | --- | --- | --- | --- |
| 2-Hydroxybenzaldehyde | 水杨醛 |  | C7H6O2 | 123.158 |
| 4-[5-(4-hydroxy-3-methoxyphenyl)-3,4-dimethyloxolan-2-yl]-2-methoxyphenol |  | phenylpropanoids(苯丙素类) | C20H24O5 | 422.714 |
| Apigenin | 芹菜素 | flavonoids(黄酮类) | C15H10O5 | 280.906 |
| Chrysin | 白杨素 | flavonoids(黄酮类) | C15H10O4 | 268.001 |
| Coumaric acid |  | phenylpropanoids(苯丙素类) | C9H8O3 | 82.7379 |
| Curcumenol | 莪术烯醇 | terpenoids(萜类) | C15H22O2 | 542.989 |
| Daidzein | 大豆苷元 | flavonoids(黄酮类) | C15H10O4 | 183.429 |
| Daidzein-8-C-glucoside |  | flavonoids(黄酮类) | C21H20O9 | 241.582 |
| Diffractic acid | 地弗地衣酸 | Phenols(酚类) | C20H22O7 | 299.833 |
| Emodin | 大黄素 | quinones(醌类) | C15H10O5 | 109.479 |
| Flavone base + 3O, 1MeO | 香叶木素 | flavonoids(黄酮类) | C16H12O6 | 252.311 |
| Gallic acid | 没食子酸 | Phenols(酚类) | C7H6O5 | 35.5468 |
| Gibberellin A19 | 赤霉素A19 | terpenoids(萜类) | C20H26O6 | 311.994 |
| Irigenin | 野鸢尾黄素 | flavonoids(黄酮类) | C18H16O8 | 215.7175 |
| Isokobusone | 异考布松 | terpenoids(萜类) | C14H22O2 | 555.284 |
| Kaempferol | 山柰酚 | flavonoids(黄酮类) | C15H10O6 | 491.741 |
| Myricitrin | 杨梅苷 | flavonoids(黄酮类) | C21H20O12 | 112.234 |
| p-Hydroxybenzaldehyde | 对羟基安息香醛 | Phenols(酚类) | C7H6O2 | 76.2581 |
| Palmitic acid | 棕榈酸； 十六酸； 软脂酸 | Aliphatic acyl(脂肪酰类) | C16H32O2 | 1.42823 |
| Scutellarein | 高黄芩素 | flavonoids(黄酮类) | C15H10O6 | 166.76 |
| Sophoricoside | 槐角苷 | flavonoids(黄酮类) | C21H20O10 | 233.189 |
| Baicalein | 黄芩素 | flavonoids(黄酮类) | C15H10O5 | 229.932 |
| Genistein | 染料木素 | flavonoids(黄酮类) | C15H10O5 | 90.9886 |
| Biochanin A | 鹰嘴豆芽素A | flavonoids(黄酮类) | C16H12O5 | 248.225 |
| 2-[4,5-Dihydroxy-6-[[8-hydroxy-8a-(hydroxymethyl)-4,4,6a,6b,11,11,14b-heptamethyl-1,2,3,4a,5,6,7,8,9,10,12,14a-dodecahydropicen-3-yl]oxy]-2-[[3,4,5-trihydroxy-6-(hydroxymethyl)oxan-2-yl]oxymethyl]oxan-3-yl]oxy-6-methyloxane-3,4,5-triol | 柴胡皂苷BK1 | terpenoids(萜类) | C48H78O17 | 418.9595 |
| Formononetin | 刺芒柄花素 | flavonoids(黄酮类) | C16H12O4 | 187.277 |
| Licochalcone-C | 甘草查尔酮C | flavonoids(黄酮类) | C21H22O4 | 439.149 |
| Enoxolone |  | terpenoids(萜类) | C30H46O4 | 602.9035 |
| 2,3-bis[(4-hydroxy-3-methoxyphenyl)methyl]butane-1,4-diol | (-)-开环异落叶松醇 | phenylpropanoids(苯丙素类) | C20H26O6 | 492.375 |
| Carpachromene | 卡帕色烯 | flavonoids(黄酮类) | C20H16O5 | 509.669 |
| Piceatannol | 白皮杉醇； 比杉特醇 | Phenols(酚类) | C14H12O4 | 118.819 |
| Flavone base + 2O+1MeO | 金合欢素 | flavonoids(黄酮类) | C16H12O5 | 267.423 |
| Genkwanin | 芫花素 | flavonoids(黄酮类) | C16H12O5 | 77.9627 |
| Mulberrin | 桑黄酮 | flavonoids(黄酮类) | C25H26O6 | 560.473 |
| Isoliquiritigenin | 异甘草素 | flavonoids(黄酮类) | C15H12O4 | 288.356 |
| Licoricesaponin G2 | 甘草皂苷G2 | terpenoids(萜类) | C42H62O17 | 305.572 |
| 3-hydroxybenzoic acid | 3-羟基苯甲酸 | Aromaticity(芳香族化合物) | C7H6O3 | 52.1227 |
| cirsimaritin | 滨蓟黄素 | flavonoids(黄酮类) | C17H14O6 | 23.5096 |
| Methyl hexadecanoate | 棕榈酸甲酯 | Aliphatic acyl(脂肪酰类) | C17H34O2 | 522.199 |
| Pyrogallol | 焦性没食子酸 | Phenols(酚类) | C6H6O3 | 37.68105 |
| Matairesinol |  | phenylpropanoids(苯丙素类) | C20H22O6 | 350.123 |
| Citric acid | 柠檬酸 | Carboxylic acids and derivatives(羧酸及其衍生物) | C6H8O7 | 33.2662 |
| FA 18:1+3O |  |  | C18H34O5 | 315.393 |
| Licoricesaponin H2 |  | terpenoids(萜类) | C42H62O16 | 258.689 |
| Piperonylic Acid | 胡椒酸 | Organic acids and derivatives(有机酸及其衍生物) | C8H6O4 | 56.8863 |
| Salicylic acid | 水杨酸 | Phenols(酚类) | C7H6O3 | 154.603 |
| Luteolin | 木犀草素 | flavonoids(黄酮类) | C15H10O6 | 287.762 |
| Eupafolin | 泽兰黄酮 | flavonoids(黄酮类) | C16H12O7 | 208.915 |
| Caffeic Acid | 咖啡酸 | phenylpropanoids(苯丙素类) | C9H8O4 | 53.6256 |
| Pectolinarigenin | 柳穿鱼黄素 | flavonoids(黄酮类) | C17H14O6 | 401.197 |
| columbianetin | 二氢山芹醇(哥伦比亚苷元) | phenylpropanoids(苯丙素类) | C14H14O4 | 283.151 |
| Ginsenoside Ro | 人参皂苷Ro | terpenoids(萜类) | C48H76O19 | 294.153 |
| Flavanone base + 3O, 1Prenyl | 甘草黄酮提取物 | flavonoids(黄酮类) | C20H20O5 | 437.205 |
| Atractylenolide III | 白术内酯Ⅲ | terpenoids(萜类) | C15H20O3 | 371.152 |
| Jaceosidin | 棕矢车菊素 | flavonoids(黄酮类) | C17H14O7 | 274.432 |
| Soyasapogenol E base + O-HexA-Hex-dHex | 脱氢皂甙 | terpenoids(萜类) | C48H76O18 | 433.838 |
| alpha-Hederin |  | terpenoids(萜类) | C41H66O12 | 408.411 |
| 5-OXO-D-PROLINE | L-焦谷氨酸 | Amino acid derivatives(氨基酸衍生物) | C5H7NO3 | 32.7697 |
| D-Gluconic acid |  | Organic acids and derivatives(有机酸及其衍生物) | C6H12O7 | 32.7697 |
| Soyasapogenol B base + O-HexA-Pen-dHex |  | terpenoids(萜类) | C47H76O17 | 407.195 |
| 1,2-Dihydroxy anthraquinone | 茜素 | quinones(醌类) | C14H8O4 | 350.123 |
| Quercetin | 槲皮素 | flavonoids(黄酮类) | C15H10O7 | 200.88 |
| Biochanin-7-O-glucoside |  | flavonoids(黄酮类) | C22H22O10 | 248.774 |
| Methyl 4-hydroxycinnamate | 4-羟基肉桂酸甲酯 | phenylpropanoids(苯丙素类) | C10H10O3 | 98.4598 |
| Licochalcone B | 甘草查尔酮B | flavonoids(黄酮类) | C16H14O5 | 184.52 |
| Neobavaisoflavone | 新补骨脂异黄酮 | flavonoids(黄酮类) | C20H18O4 | 392.852 |
| Soyasapogenol B base + O-HexA+HexA+dHex |  | terpenoids(萜类) | C48H74O19 | 377.283 |
| Icaritin | 淫羊藿素 | flavonoids(黄酮类) | C21H20O6 | 401.788 |
| Abscisic acid | 5-(1-羟基-2,6,6-三甲基-4-氧代-2-环己烯-1-基)-3-甲基-2,4-戊二烯酸 | terpenoids(萜类) | C15H20O4 | 204.94 |
| Isoferulic acid | 异阿魏酸 | phenylpropanoids(苯丙素类) | C10H10O4 | 156.882 |
| Fumaric acid | 富马酸 | Organic acids and derivatives(有机酸及其衍生物) | C4H4O4 | 32.7697 |
| Flavonol base + 3O, O-Hex |  | flavonoids(黄酮类) | C21H20O11 | 107.277 |
| Herbacetin | 草质素 | flavonoids(黄酮类) | C15H10O7 | 125.387 |
| rutamarin |  | phenylpropanoids(苯丙素类) | C21H24O5 | 772.764 |
| 5,7-dihydroxy-2-(4-hydroxyphenyl)-8-[3,4,5-trihydroxy-6-(hydroxymethyl)oxan-2-yl]-6-(3,4,5-trihydroxyoxan-2-yl)chromen-4-one | 异夏佛塔苷 | flavonoids(黄酮类) | C26H28O14 | 69.1158 |
| Gibberellin A44 | 赤霉素A44 | terpenoids(萜类) | C20H26O5 | 457.601 |
| 7-Hydroxycoumarin | 7-羟基香豆素 | phenylpropanoids(苯丙素类) | C9H6O3 | 88.8405 |
| Gentisic acid | 龙胆酸 | Xanthones(山酮类) | C7H6O4 | 64.8581 |
| Isobavachalcone | 补骨脂乙素 | flavonoids(黄酮类) | C20H20O4 | 475.878 |
| Orcinol | 苔黑酚葡萄糖苷 | Phenols(酚类) | C7H8O2 | 60.6486 |
| Asiaticoside | 积雪草苷 | terpenoids(萜类) | C48H78O19 | 493.654 |
| Benzoic acid + 2O, O-Hex | 苯甲酸+2O，O-六角 |  | C13H16O9 | 35.5468 |
| Vanillin | 香草醛 | Phenols(酚类) | C8H8O3 | 87.1296 |
| Rheic acid | 大黄酸 | quinones(醌类) | C15H8O6 | 350.123 |
| Flavone base + 3O, C-Hex-dHex |  | flavonoids(黄酮类) | C27H30O14 | 83.831 |
| Sebacic acid | 癸二酸 | Aliphatic acyl(脂肪酰类) | C10H18O4 | 211.106 |
| Isoanhydroicaritin | 异脱水淫羊霍素 | flavonoids(黄酮类) | C21H20O6 | 352.486 |
| 2',4'-DIHYDROXYACETOPHENONE | 2’，4’-二羟基苯乙酮 | Aromaticity(芳香族化合物) | C8H8O3 | 59.2674 |
| Zingerone | 姜酮 |  | C11H14O3 | 188.955 |
| 9-Hydroxy-10,12,15-octadecatrienoic acid |  |  | C18H30O3 | 518.902 |
| Methylparaben | 尼泊金甲 | Phenols(酚类) | C8H8O3 | 39.4172 |
| Xanthyletin | 花椒内酯 | phenylpropanoids(苯丙素类) | C14H12O3 | 244.792 |
| Glutaric acid | 2-(羟基亚胺)戊烷二酸 | Organic acids and derivatives(有机酸及其衍生物) | C5H8O4 | 772.182 |
| Quercetin-3-O-galactoside | 金丝桃苷(紫花杜鹃素丁;槲皮素-3-O-β-D-半乳糖苷) | flavonoids(黄酮类) | C21H20O12 | 156.882 |
| (+/-)-Jasmonic acid | 茉莉酸 | Fatty acids(脂肪酸类) | C12H18O3 | 315.973 |
| P-Anisic acid | 对甲氧基苯甲酸 | Phenols(酚类) | C8H8O3 | 933.7415 |
| alpha,beta-Dihydroresveratrol | α，β-二氢白藜芦醇 | Phenols(酚类) | C14H14O3 | 392.268 |
| Haematoxylin | 苏木精 | phenylpropanoids(苯丙素类) | C16H14O6 | 138.551 |
| PHENYLACETIC ACID | 苯乙酸 | Aromaticity(芳香族化合物) | C8H8O2 | 90.9886 |
| Piscidic Acid |  |  | C11H12O7 | 37.0057 |
| Dihydrokaempferol | 二氢山柰酚 | flavonoids(黄酮类) | C15H12O6 | 193.312 |
| Azelaic acid | 壬二酸 | Aliphatic acyl(脂肪酰类) | C9H16O4 | 152.386 |
| Flavone base + 3O, O-Hex-Pen |  | flavonoids(黄酮类) | C26H28O14 | 57.9185 |
| 5,7-dihydroxy-2-(4-hydroxyphenyl)-6,8-bis[3,4,5-trihydroxy-6-(hydroxymethyl)oxan-2-yl]chromen-4-one |  | flavonoids(黄酮类) | C27H30O15 | 42.7394 |
| Emodin-3-methyl ether/Physcion | 大黄素甲醚 | quinones(醌类) | C16H12O5 | 435.138 |
| Inositol | 肌醇 |  | C6H12O6 | 32.4335 |
| Hydroxyferulic acid | 5-羟基阿魏酸 | phenylpropanoids(苯丙素类) | C10H10O5 | 43.9702 |
| kojic acid | 曲酸 |  | C6H6O4 | 16.2451 |
| Goniothalenol | 哥纳香醇 | phenylpropanoids(苯丙素类) | C13H12O4 | 136.979 |
| Methyl Heptadecanoic acid | 甲基七烷酸 | Aliphatic acyl(脂肪酰类) | C18H36O2 | 3.44906 |
| 5,9-dihydroxy-5,7,7-trimethyl-4,5a,6,8,8a,9-hexahydro-1H-azuleno[5,6-c]furan-3-one |  | terpenoids(萜类) | C15H22O4 | 324.346 |
| 1,7-bis(4-hydroxyphenyl)heptan-3-one | 槭苷元G | Phenols(酚类) | C19H22O3 | 531.955 |
| Demethylwedelolactone | 去甲蟛蜞菊内酯 | phenylpropanoids(苯丙素类) | C15H8O7 | 293.067 |
| Pinocembrin | 松属素 | flavonoids(黄酮类) | C15H12O4 | 116.815 |
| Suberic acid | 辛二酸 | Fatty acids(脂肪酸类) | C8H14O4 | 94.5417 |
| 2-METHYLMALEATE | 2-马来酸甲酯 | Aliphatic acyl(脂肪酰类) | C5H6O4 | 39.3634 |
| Homogentisic acid | 同质酸 | Phenols(酚类) | C8H8O4 | 60.6486 |
| Zizyberanalic acid |  | terpenoids(萜类) | C42H64O16 | 397.099 |
| Flavanone + 3O, O-Hex | 柚皮素-7-O-葡萄糖甙 | flavonoids(黄酮类) | C21H22O10 | 86.35545 |
| Syringic acid | 丁香酸 | Phenols(酚类) | C9H10O5 | 204.36 |
| Naringenin chalcone | 柚皮苷查尔酮 | flavonoids(黄酮类) | C15H12O5 | 315.4 |
| Phloretin-2'-O-glucoside | 根皮苷 | flavonoids(黄酮类) | C21H24O10 | 161.093 |
| Kaempferol-3-O-glucuronoside |  | flavonoids(黄酮类) | C21H18O12 | 122.582 |
| Aurantio-obtusin | 橙黄决明素 | quinones(醌类) | C17H14O7 | 192.733 |
| gardenoside | 山栀子苷B | terpenoids(萜类) | C17H24O11 | 65.4555 |
| Ethyl caffeate | 咖啡酸乙酯 | phenylpropanoids(苯丙素类) | C11H12O4 | 246.028 |
| Eupatilin | 异泽兰黄素 | flavonoids(黄酮类) | C18H16O7 | 335.869 |
| Petroselinic acid | 十八碳6烯酸 |  | C18H34O2 | 705.604 |
| Soybean saponin fraction B1 | 大豆皂苷 | terpenoids(萜类) | C48H78O18 | 393.441 |
| GALACTARATE | 半乳糖 | Carbohydrates and derivatives(糖类及其衍生物) | C6H10O8 | 36.0882 |
| Swertiamarin | 獐牙菜苦苷 | terpenoids(萜类) | C16H22O10 | 37.4306 |
| Wedelolactone | 蟛蜞菊内酯 | phenylpropanoids(苯丙素类) | C16H10O7 | 267.423 |
| Azuleno(5,6-c)furan-1(3H)-one, 4,4a,5,6,7,7a,8,9-octahydro-3,4,8-trihydroxy-6,6,8-trimethyl- |  | terpenoids(萜类) | C15H22O5 | 307.6505 |
| Oleanane -2H, +1O, 1COOH, O-HexA-HexA |  | terpenoids(萜类) | C42H64O15 | 377.554 |
| Quercetin-3-O-glucuronide | 槲皮素-3-葡萄糖醛酸苷 | flavonoids(黄酮类) | C21H18O13 | 83.2693 |
| Benzyl alcohol + Hex-Pen |  |  | C18H26O10 | 56.8863 |
| Isoflavone base + 2O, 1MeO | 黄豆黄素 | flavonoids(黄酮类) | C16H12O5 | 281.48 |
| Ginkgolide A | 银杏内酯A | terpenoids(萜类) | C20H24O9 | 231.026 |
| Casticin | 蔓荆子黄素 | flavonoids(黄酮类) | C19H18O8 | 333.564 |
| 3',5'-Dimethoxy-4'-hydroxyacetophenone | 乙酰丁香酮 | Phenols(酚类) | C10H12O4 | 3.44906 |
| Demethoxycurcumin | 去甲氧基姜黄素 | Phenols(酚类) | C20H18O5 | 380.439 |
| Wikstromol | (-)-亚洲络石脂内酯 | phenylpropanoids(苯丙素类) | C20H22O7 | 289.532 |
| tectorigenin |  | flavonoids(黄酮类) | C16H12O6 | 183.616 |
| Liquiritin | 甘草苷 | flavonoids(黄酮类) | C21H22O9 | 116.815 |
| 1,3-Dicaffeoylquinic acid | 1,3-二咖啡酰奎宁酸 | phenylpropanoids(苯丙素类) | C25H24O12 | 50.06 |
| Flavone base + 2O, 1MeO, C-Hex |  | flavonoids(黄酮类) | C22H22O10 | 77.3901 |
| SUCROSE | 蔗糖 | Carbohydrates and derivatives(糖类及其衍生物) | C12H22O11 | 32.4335 |
| 3,4,5-trimethoxycinnamic acid | 3,4,5-三甲氧基肉桂酸 | phenylpropanoids(苯丙素类) | C12H14O5 | 76.2581 |
| Echinocystic acid-3-O-glucoside | 七叶皂甙 | terpenoids(萜类) | C36H58O9 | 450.61 |
| Adipic acid | 己二酸 | Fatty acids(脂肪酸类) | C6H10O4 | 741.76 |
| (EZ)-sinapic acid | 芥子酸 | phenylpropanoids(苯丙素类) | C11H12O5 | 123.736 |
| 6-Prenylnaringenin | 6-戊基柚皮苷 | flavonoids(黄酮类) | C20H20O5 | 385.682 |
| Diethyl-phthalate | 邻苯二甲酸二乙酯 | Aromaticity(芳香族化合物) | C12H14O4 | 226.322 |
| (E/Z)-cinnamic acid | 肉桂酸 | phenylpropanoids(苯丙素类) | C9H8O2 | 149.264 |
| Vanillic acid | 香草酸 | Organic acids and derivatives(有机酸及其衍生物) | C8H8O4 | 39.87255 |
| 4-Methoxysalicylic acid | 4-甲氧基水杨酸 | Phenols(酚类) | C8H8O4 | 81.7563 |
| Desmethylxanthohumol |  | flavonoids(黄酮类) | C20H20O5 | 405.318 |
| Formononetin-7-O-glucoside |  | flavonoids(黄酮类) | C22H22O9 | 190.005 |
| skullcapflavone II |  | flavonoids(黄酮类) | C19H18O8 | 120.897 |
| Pimelic acid | 庚二酸 | Fatty acids(脂肪酸类) | C7H12O4 | 60.6486 |
| 3-(2-HYDROXYPHENYL)PROPANOATE | 3-（2-羟基苯基）丙酸盐 |  | C9H10O3 | 48.04645 |
| 4-[2-(2,6-dimethoxy-4-prop-2-enylphenoxy)-1-hydroxypropyl]-2-methoxyphenol |  |  | C21H26O6 | 311.0545 |
| Ingenol | 巨大戟醇 | terpenoids(萜类) | C20H28O5 | 380.454 |
| Cinnamic acid | 肉桂酸 | phenylpropanoids(苯丙素类) | C9H8O2 | 119.132 |
| Picrotin | 苦亭 | terpenoids(萜类) | C15H18O7 | 149.264 |
