# Supplementary material for: Integrated network pharmacology and experimental verification to reveal the role of Shezhi Huangling Decoction against glioma by inactivating PI3K/Akt-HIF1A axis
Source: Heliyon. 2024 Jul 6;10(14):e34215. doi: 10.1016/j.heliyon.2024.e34215 (PMC11292238; doi:10.1016/j.heliyon.2024.e34215)
Supplement: Multimedia component 3 [file mmc3.doc]

**Table S3 overlapping genes between brain glioma-related target genes and active compounds of SHD related target genes**

| **Number** | **Protein name** | **Gene name** |
| --- | --- | --- |
| 1 | Protein tyrosine phosphatase non-receptor type 11 | PTPN11 |
| 2 | Vascular endothelial growth factor A | VEGFA |
| 3 | Isocitrate dehydrogenase (NADP(+)) 1 | IDH1 |
| 4 | Signal transducer and activator of transcription 3 | STAT3 |
| 5 | Cyclin dependent kinase 6 | CDK6 |
| 6 | Paired box 6 | PAX6 |
| 7 | ATM serine/threonine kinase | ATM |
| 8 | NF2, moesin-ezrin-radixin like (MERLIN) tumor suppressor | NF2 |
| 9 | Smoothened, frizzled class receptor | SMO |
| 10 | BCL2 apoptosis regulator | BCL2 |
| 11 | B-Raf proto-oncogene, serine/threonine kinase | BRAF |
| 12 | Interferon gamma | IFNG |
| 13 | Catenin beta 1 | CTNNB1 |
| 14 | Contactin 2 | CNTN2 |
| 15 | Superoxide dismutase 1 | SOD1 |
| 16 | Nitric oxide synthase 2 | NOS2 |
| 17 | Dual specificity tyrosine phosphorylation regulated kinase 1A | DYRK1A |
| 18 | Rac family small GTPase 1 | RAC1 |
| 19 | Epidermal growth factor receptor | EGFR |
| 20 | Protection of telomeres 1 | POT1 |
| 21 | Hypoxia inducible factor 1 subunit alpha | HIF1A |
| 22 | Kinase insert domain receptor | KDR |
| 23 | Telomerase reverse transcriptase | TERT |
| 24 | Tumor protein p53 | TP53 |
| 25 | Erb-b2 receptor tyrosine kinase 2 | ERBB2 |
| 26 | Phosphatidylinositol-4,5-bisphosphate 3-kinase catalytic subunit alpha | PIK3CA |
| 27 | Fibroblast growth factor receptor 1 | FGFR1 |
| 28 | BCL2 associated X, apoptosis regulator | BAX |
| 29 | Choline O-acetyltransferase | CHAT |
| 30 | Glutathione S-transferase pi 1 | GSTP1 |
| 31 | AKT serine/threonine kinase 1 | AKT1 |
| 32 | Peroxisome proliferator activated receptor gamma | PPARG |
| 33 | MutS homolog 2 | MSH2 |
