# Supplementary material for: Integrated network pharmacology and experimental verification to reveal the role of Shezhi Huangling Decoction against glioma by inactivating PI3K/Akt-HIF1A axis
Source: Heliyon. 2024 Jul 6;10(14):e34215. doi: 10.1016/j.heliyon.2024.e34215 (PMC11292238; doi:10.1016/j.heliyon.2024.e34215)
Supplement: Multimedia component 4 [file mmc4.doc]

**Table S4 Topological structure properties of PPI network**

| Genes | Betweenness Centrality | Closeness Centrality | Degree |
| --- | --- | --- | --- |
| AKT1 | 0.08275978 | 0.84210526 | 27 |
| TP53 | 0.09371817 | 0.84210526 | 26 |
| CTNNB1 | 0.08199479 | 0.8 | 24 |
| STAT3 | 0.049454 | 0.72727273 | 22 |
| EGFR | 0.0315466 | 0.72727273 | 22 |
| VEGFA | 0.02762826 | 0.74418605 | 22 |
| PIK3CA | 0.01962562 | 0.71111111 | 21 |
| ERBB2 | 0.01844176 | 0.71111111 | 21 |
| HIF1A | 0.02506555 | 0.71111111 | 20 |
| TERT | 0.03997248 | 0.68085106 | 19 |
| ATM | 0.03211977 | 0.65306122 | 17 |
| KDR | 0.01520612 | 0.66666667 | 16 |
| IDH1 | 0.00980973 | 0.65306122 | 16 |
| PTPN11 | 0.00426536 | 0.64 | 16 |
| PPARG | 0.02100145 | 0.65306122 | 15 |
| FGFR1 | 0.01254736 | 0.65306122 | 15 |
| IFNG | 0.00215141 | 0.60377358 | 13 |
| SOD1 | 0.05150212 | 0.59259259 | 12 |
| RAC1 | 0.00509628 | 0.58181818 | 11 |
| NOS2 | 0.00248198 | 0.57142857 | 10 |
| NF2 | 7.85E-04 | 0.57142857 | 10 |
| CDK6 | 1.34E-04 | 0.57142857 | 10 |
| BRAF | 3.51E-04 | 0.56140351 | 9 |
| PAX6 | 0.08565647 | 0.56140351 | 8 |
| GSTP1 | 0.00161045 | 0.54237288 | 7 |
| MSH2 | 0 | 0.54237288 | 7 |
| BCL2 | 0.00107527 | 0.53333333 | 6 |
| BAX | 3.36E-04 | 0.52459016 | 6 |
| SMO | 0.0014881 | 0.52459016 | 4 |
| DYRK1A | 5.88E-04 | 0.50793651 | 3 |
| CHAT | 0.00134409 | 0.41558442 | 2 |
| POT1 | 0 | 0.42666667 | 2 |
| CNTN2 | 0 | 0.36363636 | 1 |
