# Supplementary material for: Integrated network pharmacology and experimental verification to reveal the role of Shezhi Huangling Decoction against glioma by inactivating PI3K/Akt-HIF1A axis
Source: Heliyon. 2024 Jul 6;10(14):e34215. doi: 10.1016/j.heliyon.2024.e34215 (PMC11292238; doi:10.1016/j.heliyon.2024.e34215)
Supplement: Multimedia component 5 [file mmc5.doc]

**Table S5 Hub genes correspond to the active compounds in SHD**

| **Hub genes** | **Active compounds** |
| --- | --- |
| AKT1 | Quercetin  Dinatin  6-Hydroxynaringenin  Rhamnazin  Eriodictyol  Luteolin  Naringenin  Diosgenin  Mairin  Farnesylacetone |
| TP53 | Diosgenin  Isorhamnetin |
| CTNNB1 | Bifendate  Palmidin A |
| STAT3 | 1. methoxy-3-methyl-9,10-anthraquinone   Daucostero  Epiberberine  Farnesylacetone |
| EGFR | 2,3-dimethoxy-6-methyanthraquinone  2-methoxy-3-methyl-9,10-anthraquinone  quercetin  5,7,4'-trihydroxy-8-methoxyflavanone  5-hydroxy-7,8-dimethoxy-2-(4-methoxyphenyl)chromone  Baicalin  Rivularin  Wogonin  Dinatin  Baicalein  Salvigenin  Rhamnazin  Luteolin  Moslosooflavone  formononetin  3'-Methoxydaidzein  Daidzein  2,7-Dihydroxynaphthalene  Metilox  4-Methylcumarin |
| VEGFA | (2R)-5,7-dihydroxy-2-(4-hydroxyphenyl)chroman-4-one  Daucostero  Diosgenin  Bifendate  Calycosin  Physciondiglucoside  aloe-emodin |
| PIK3CA | 2,3-dimethoxy-6-methyanthraquinone  Rivularin  14-acetyl-12-senecioyl-2E,8Z,10E-atractylentriol  Berberine  (R)-Canadine  Berlambine  Monohexyl Phthalate  Santalol |
| ERBB2 | 3,9-di-O-methylnissolin  (6aR,11aR)-9,10-dimethoxy-6a,11a-dihydro-6H-benzofurano[3,2-c]chromen-3-ol  1,7-Dihydroxy-3,9-dimethoxy pterocarpene  Epiberberine |
| HIF1A | Eriodictyol  Diosgenin |
