# Supplementary material for: Integrated network pharmacology and experimental verification to reveal the role of Shezhi Huangling Decoction against glioma by inactivating PI3K/Akt-HIF1A axis
Source: Heliyon. 2024 Jul 6;10(14):e34215. doi: 10.1016/j.heliyon.2024.e34215 (PMC11292238; doi:10.1016/j.heliyon.2024.e34215)
Supplement: Multimedia component 6 [file mmc6.docx]

**Figure S1 The unedited images for the Figure 5D**

**HS683**

**Bax**

**
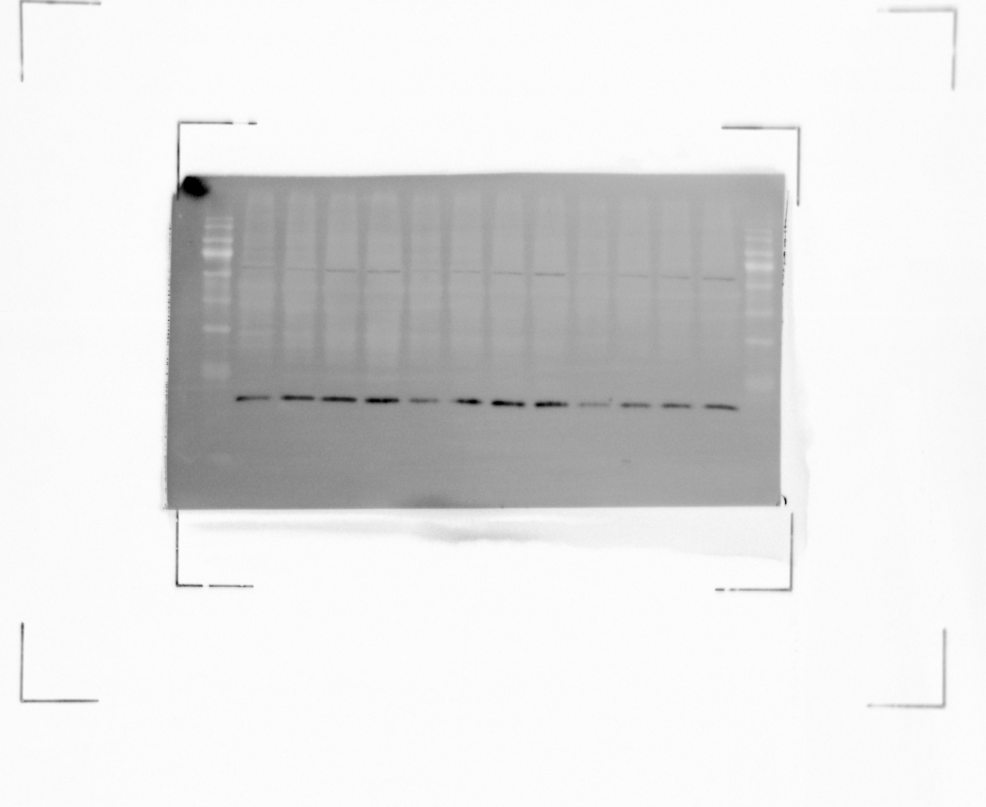
**

-100 kDa

-135 kDa

-63 kDa

-35 kDa

-17 kDa

-180 kDa

-25 kDa

-75 kDa

-48 kDa

20

Control

**SHD**

10

5

20

Control

**SHD**

10

5

20

Control

**SHD**

10

5

**Bcl-2**

**
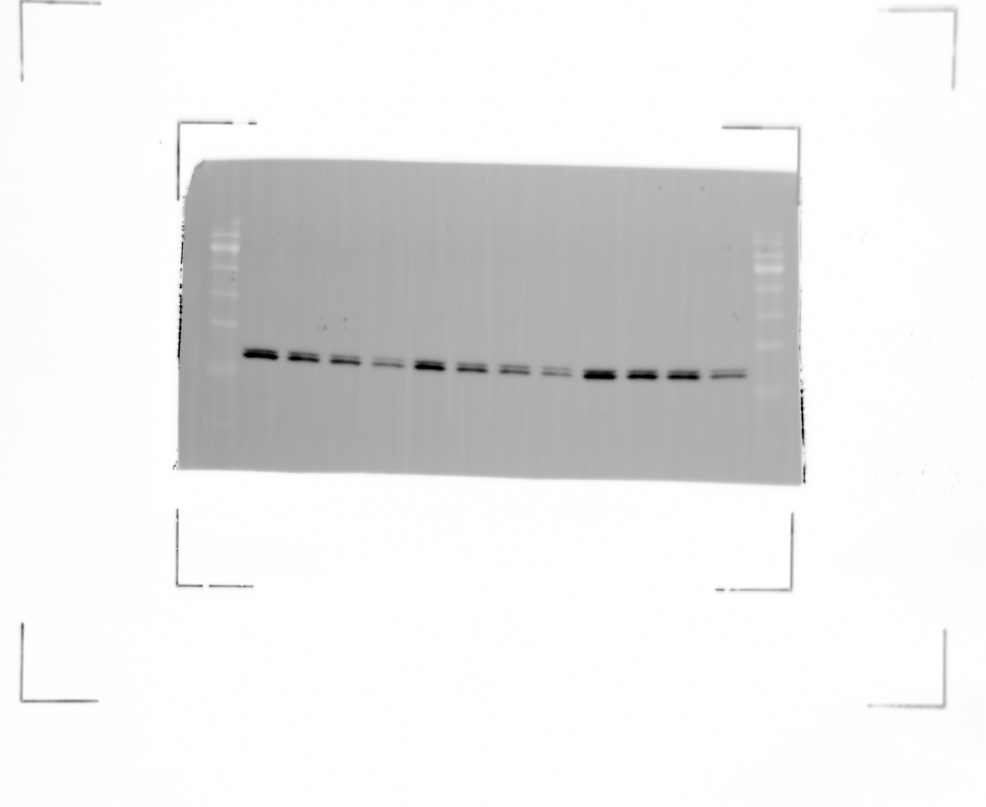
**

20

Control

**SHD**

10

5

20

Control

**SHD**

10

5

20

Control

**SHD**

10

5

**Caspase-3**

**
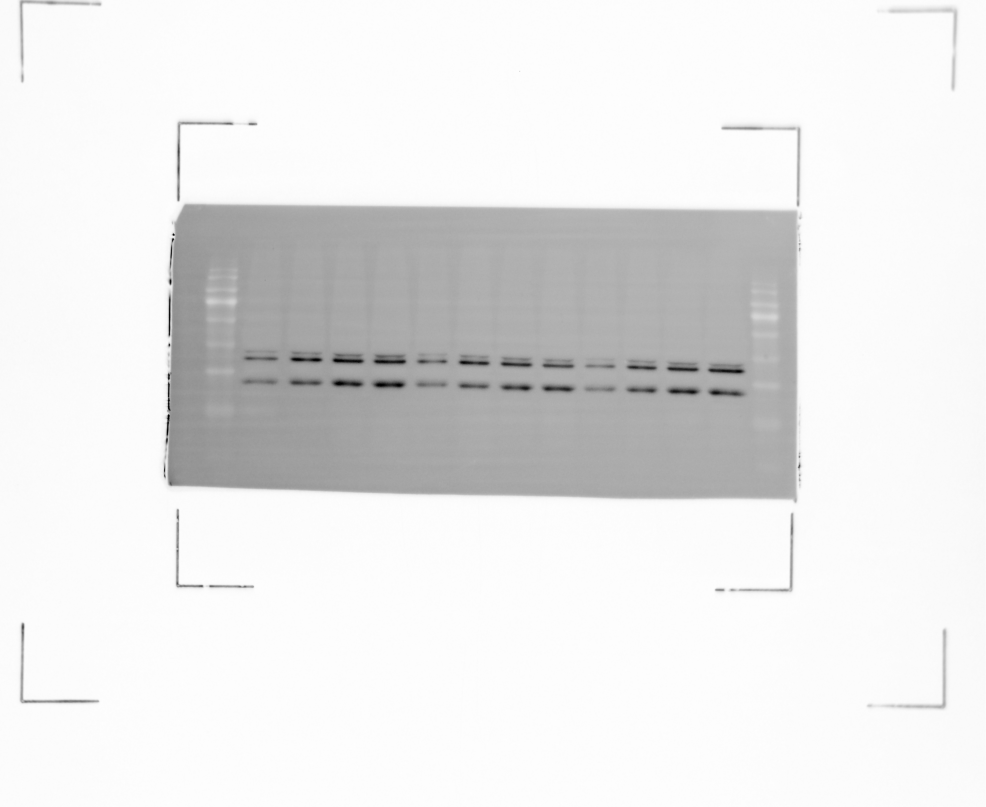
**

20

Control

**SHD**

10

5

20

Control

**SHD**

10

5

20

Control

**SHD**

10

5

**β-actin**

**
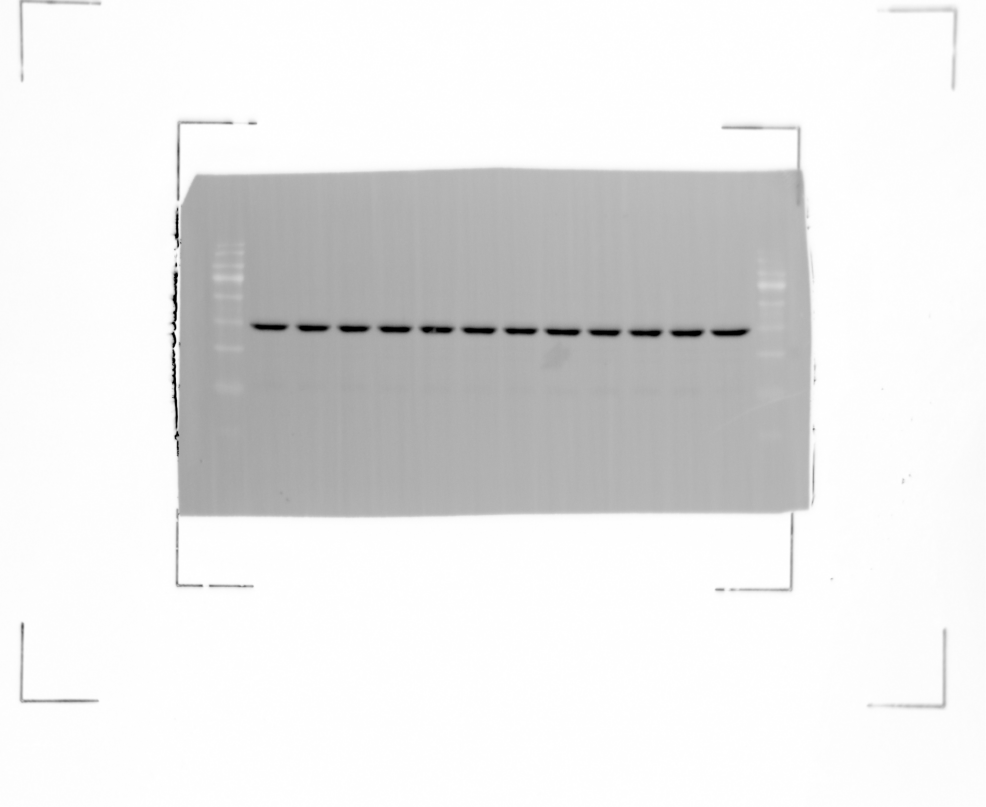
**

20

Control

**SHD**

10

5

20

Control

**SHD**

10

5

20

Control

**SHD**

10

5

**KNS89**

**Bax**

**
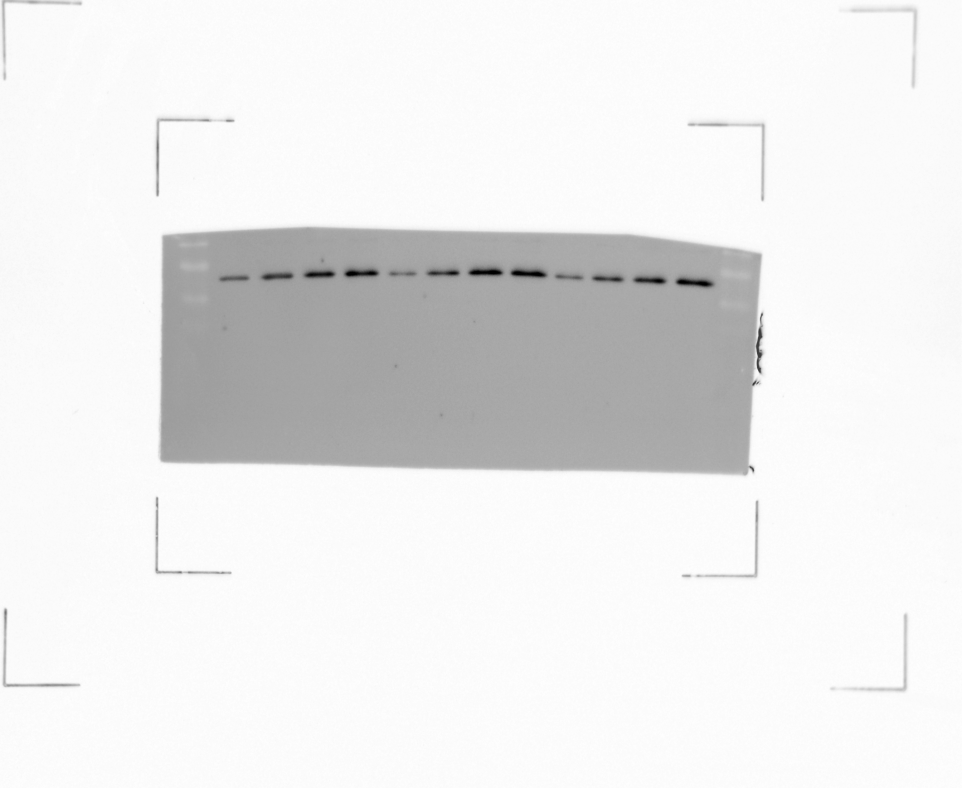
**

20

Control

**SHD**

10

5

20

Control

**SHD**

10

5

20

Control

**SHD**

10

5

**Bcl-2**

**
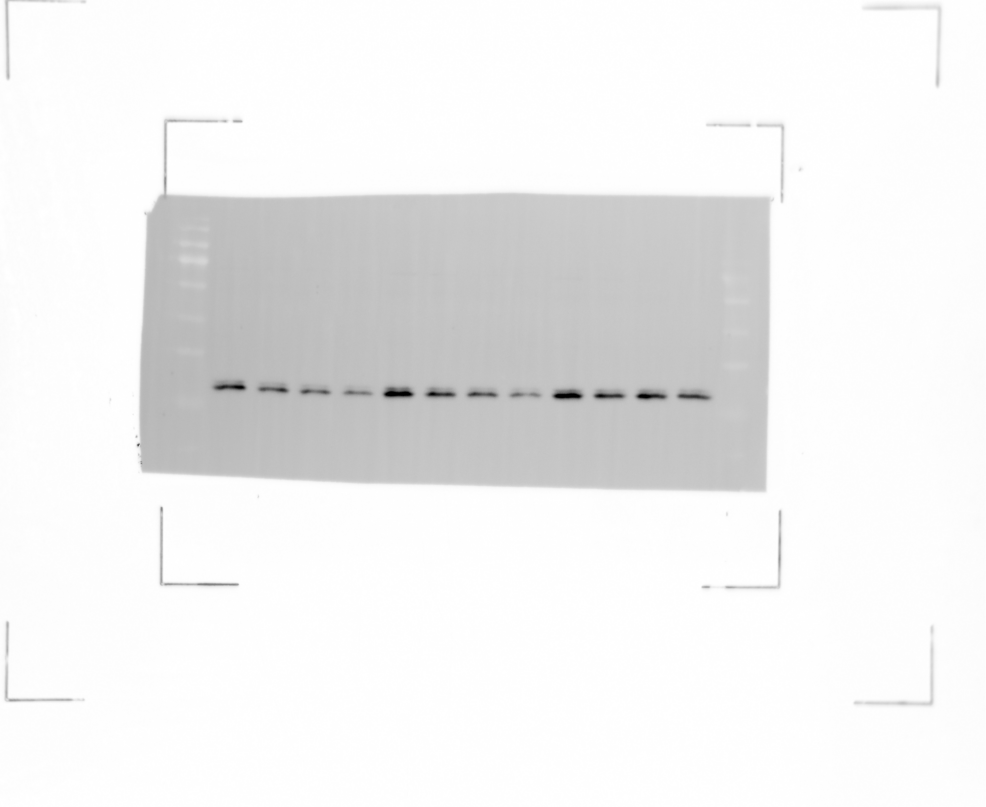
**

20

Control

**SHD**

10

5

20

Control

**SHD**

10

5

20

Control

**SHD**

10

5

**Caspase-3**

**
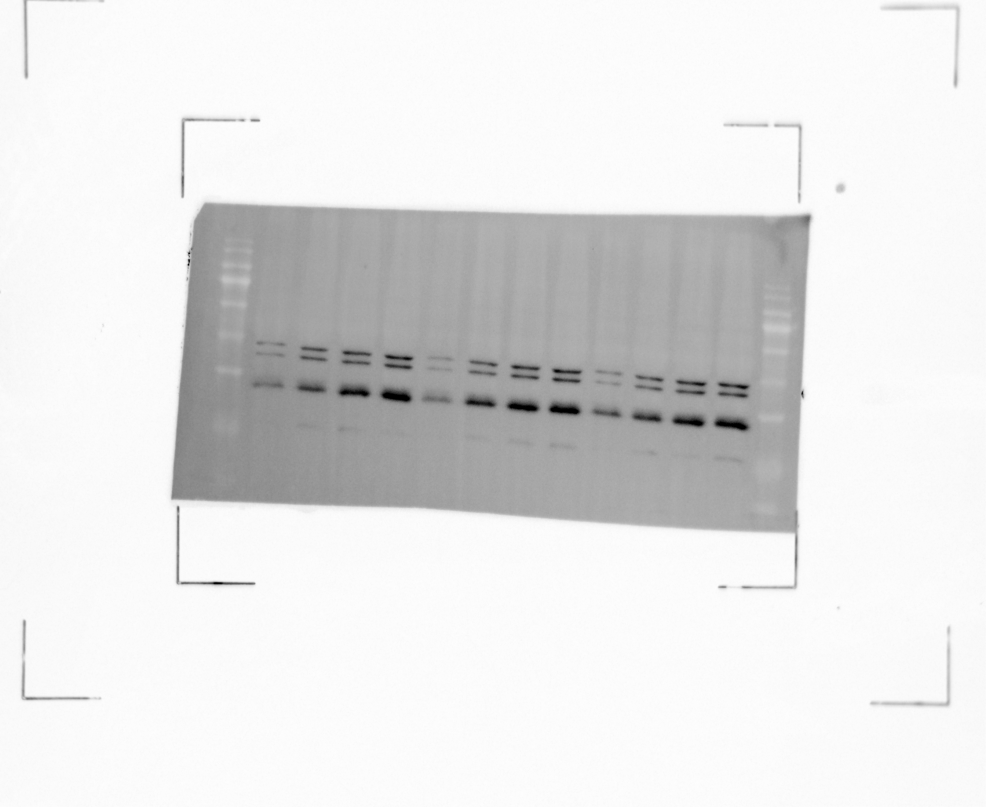
**

-100 kDa

-135 kDa

-63 kDa

-35 kDa

-17 kDa

-180 kDa

-25 kDa

-75 kDa

-48 kDa

20

Control

**SHD**

10

5

20

Control

**SHD**

10

5

20

Control

**SHD**

10

5

**β-actin**

**
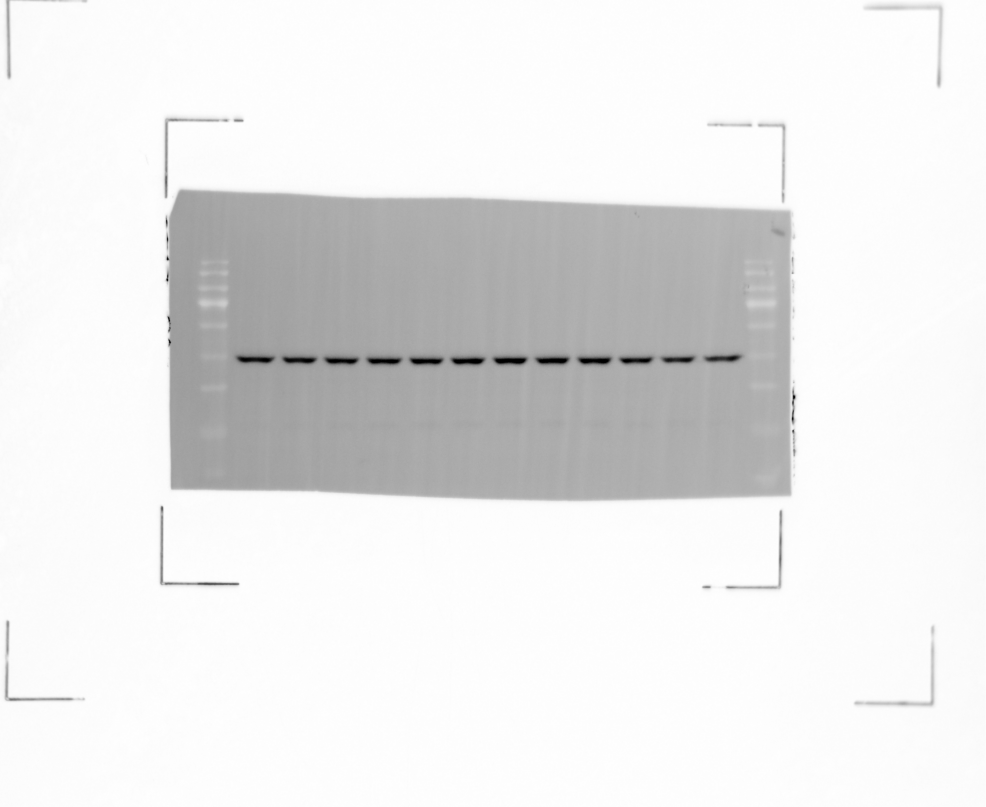
**

20

Control

**SHD**

10

5

20

Control

**SHD**

10

5

20

Control

**SHD**

10

5
