# Supplementary material for: Integrated network pharmacology and experimental verification to reveal the role of Shezhi Huangling Decoction against glioma by inactivating PI3K/Akt-HIF1A axis
Source: Heliyon. 2024 Jul 6;10(14):e34215. doi: 10.1016/j.heliyon.2024.e34215 (PMC11292238; doi:10.1016/j.heliyon.2024.e34215)
Supplement: Multimedia component 9 [file mmc9.docx]

**Figure S4 The unedited images for the Figure 8C**

**Bax**

**
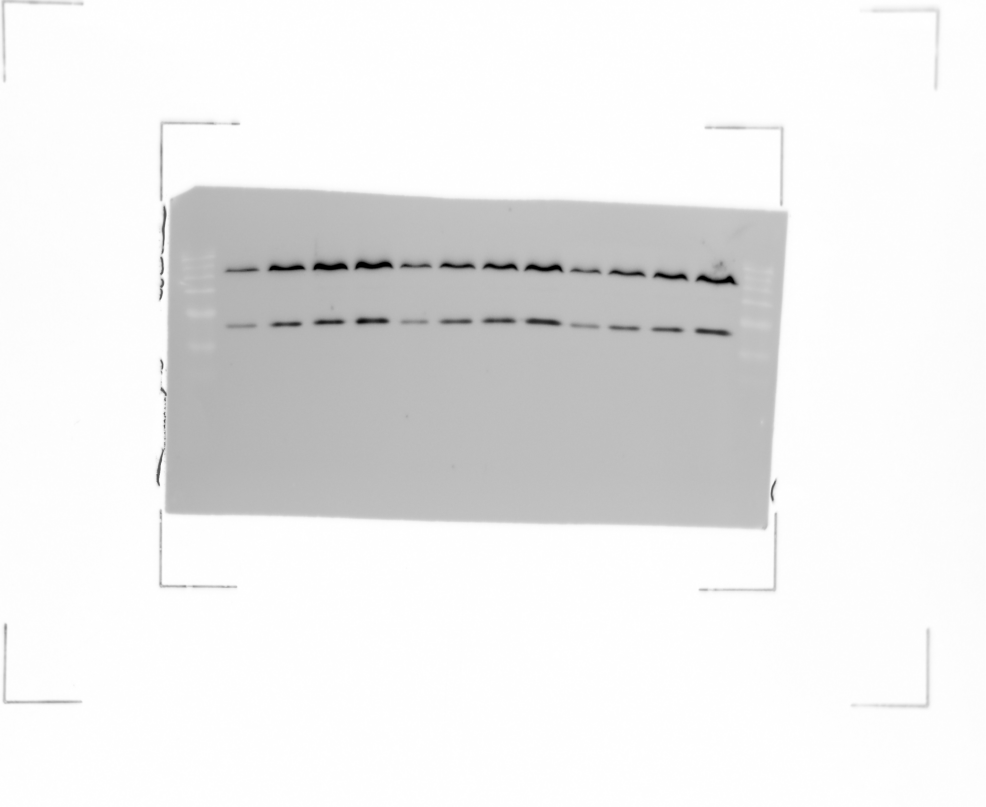
**

SHD+TMZ

Model

TMZ

SHD

SHD+TMZ

Model

TMZ

SHD

SHD+TMZ

Model

TMZ

SHD

**Bcl-2**

**
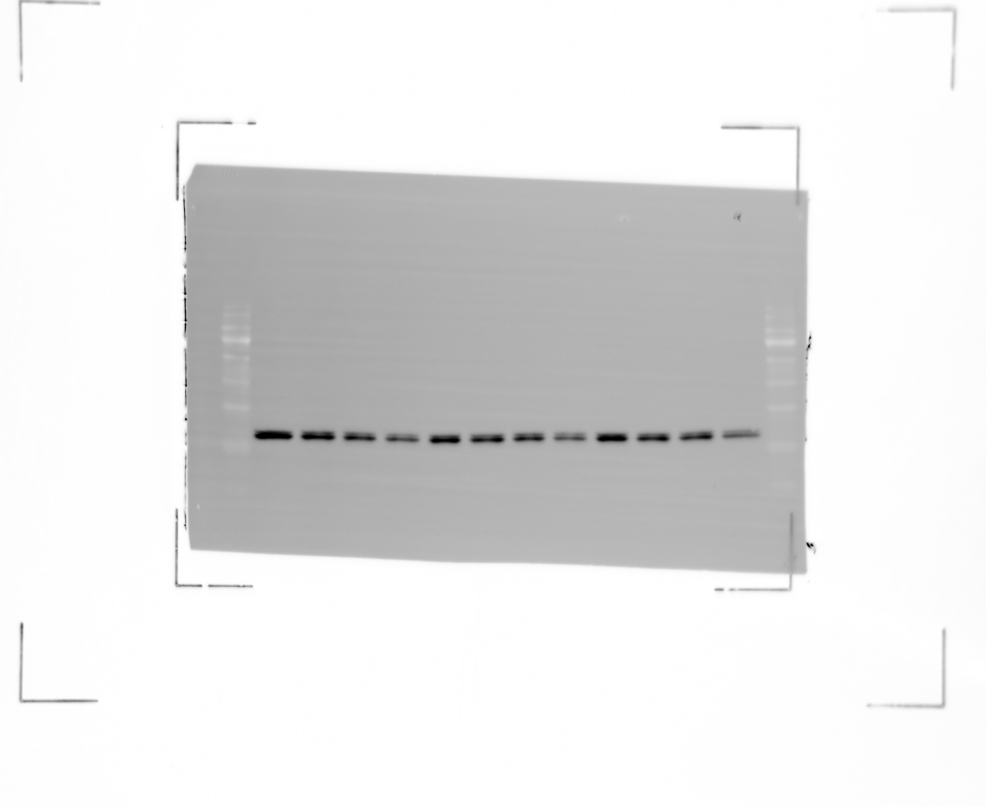
**

SHD+TMZ

Model

TMZ

SHD

SHD+TMZ

Model

TMZ

SHD

SHD+TMZ

Model

TMZ

SHD

**Caspase-3**

**
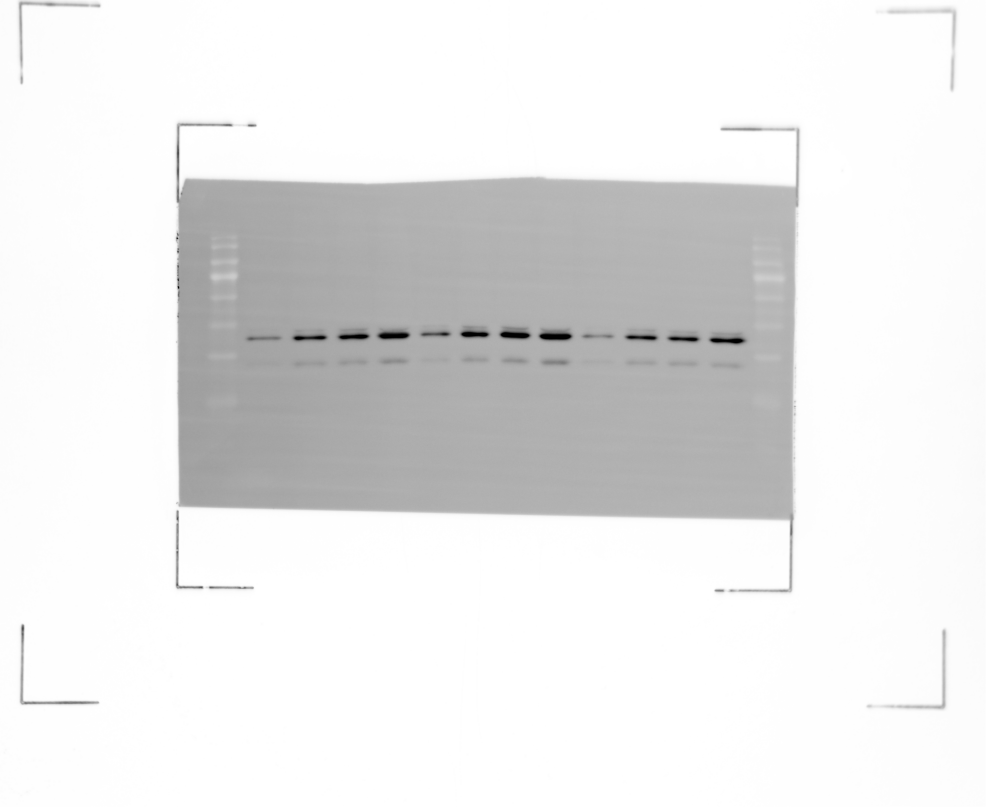
**

SHD+TMZ

Model

TMZ

SHD

SHD+TMZ

Model

TMZ

SHD

SHD+TMZ

Model

TMZ

SHD

**β-actin**

**
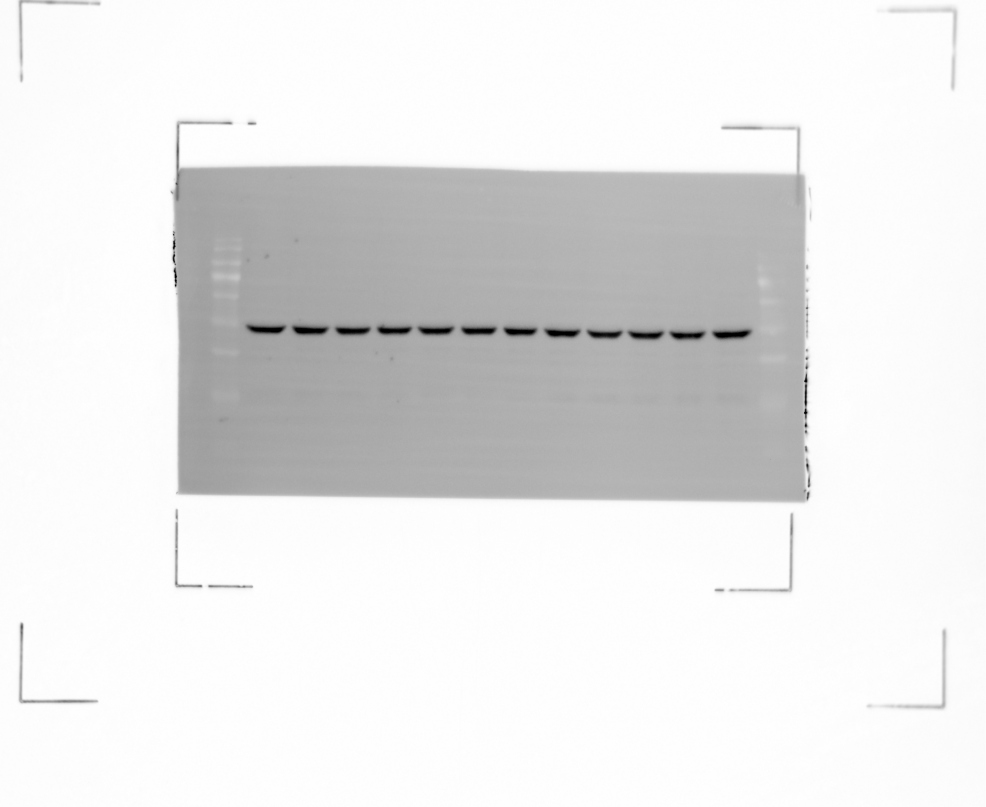
**

SHD+TMZ

Model

TMZ

SHD

SHD+TMZ

Model

TMZ

SHD

SHD+TMZ

Model

TMZ

SHD

1. **cadherin**

**
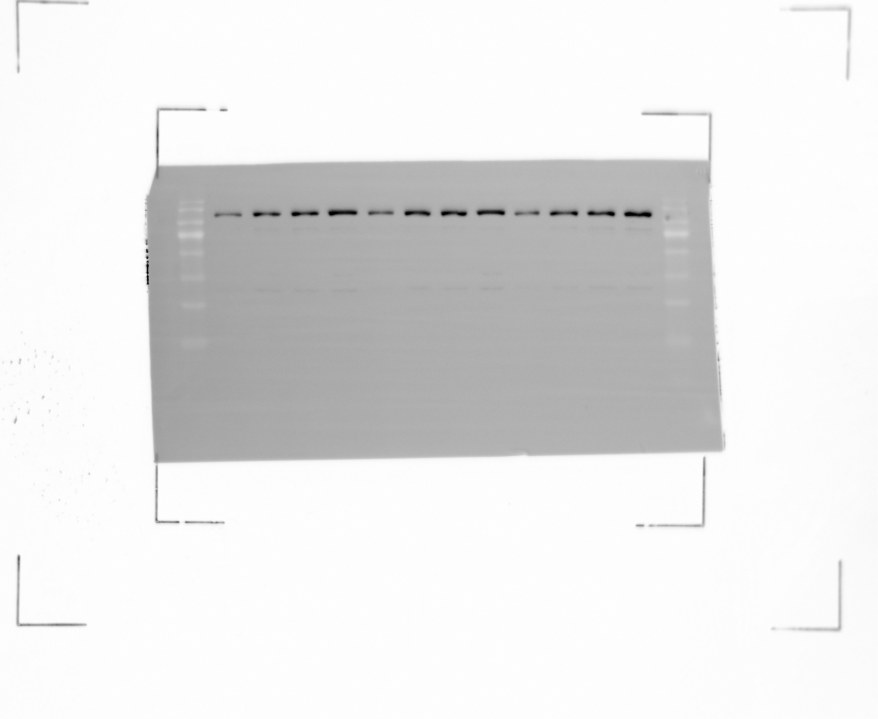
**

-100 kDa

-135 kDa

-63 kDa

-35 kDa

-17 kDa

-180 kDa

-25 kDa

-75 kDa

-48 kDa

SHD+TMZ

Model

TMZ

SHD

SHD+TMZ

Model

TMZ

SHD

SHD+TMZ

Model

TMZ

SHD

**Vimentin**

**
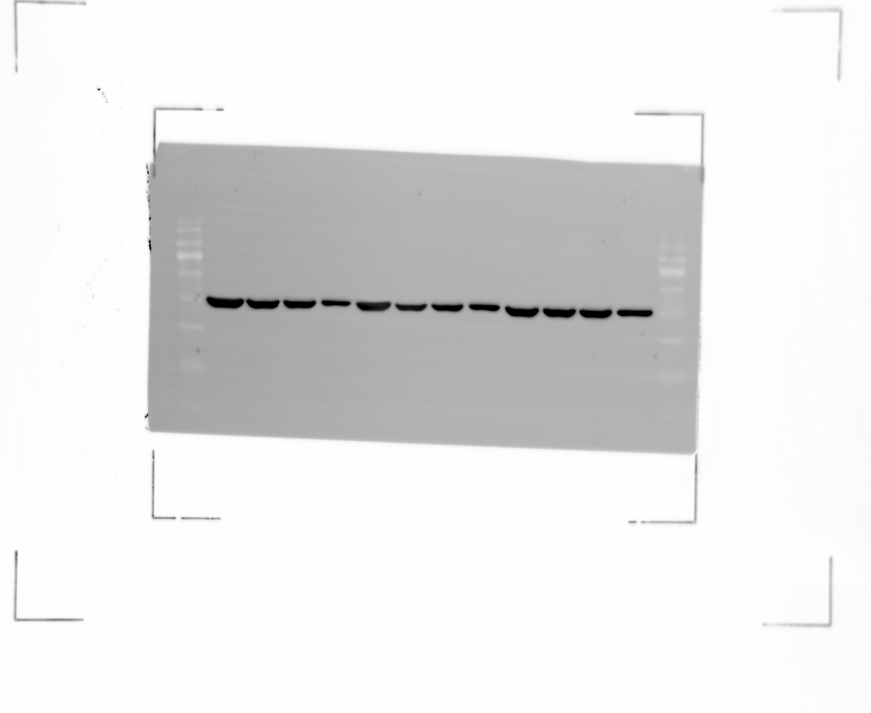
**

SHD+TMZ

Model

TMZ

SHD

SHD+TMZ

Model

TMZ

SHD

SHD+TMZ

Model

TMZ

SHD

**β-actin**

**
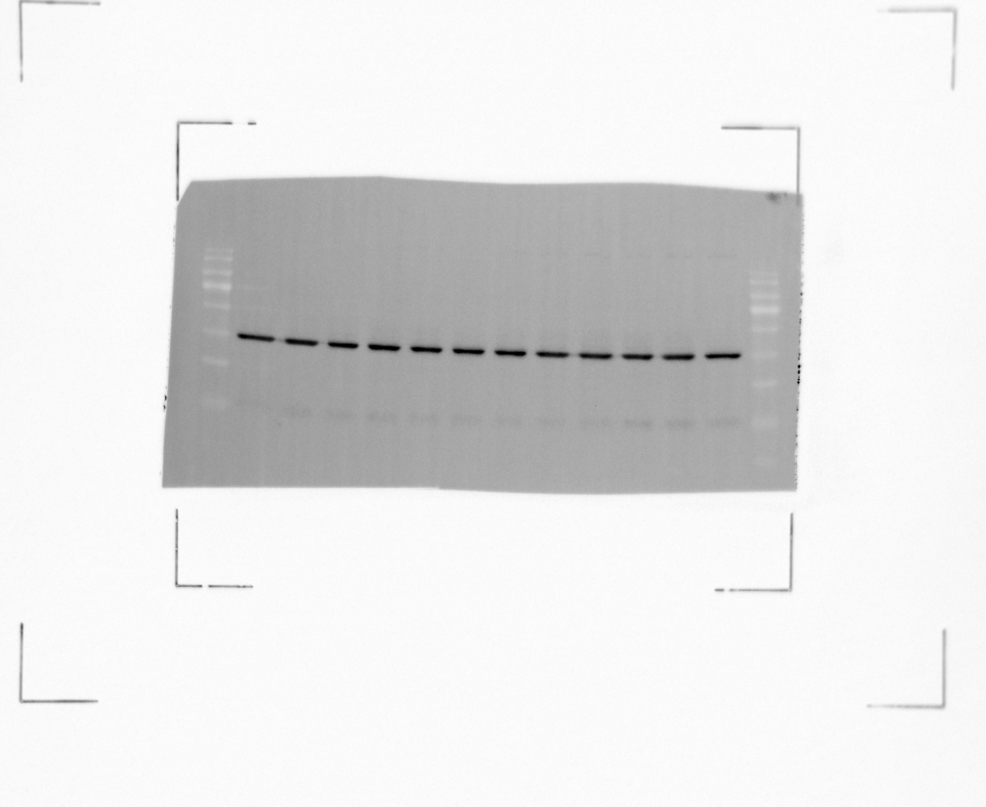
**

-100 kDa

-135 kDa

-63 kDa

-35 kDa

-17 kDa

-180 kDa

-25 kDa

-75 kDa

-48 kDa

SHD+TMZ

Model

TMZ

SHD

SHD+TMZ

Model

TMZ

SHD

SHD+TMZ

Model

TMZ

SHD

**PIK3CA**

**
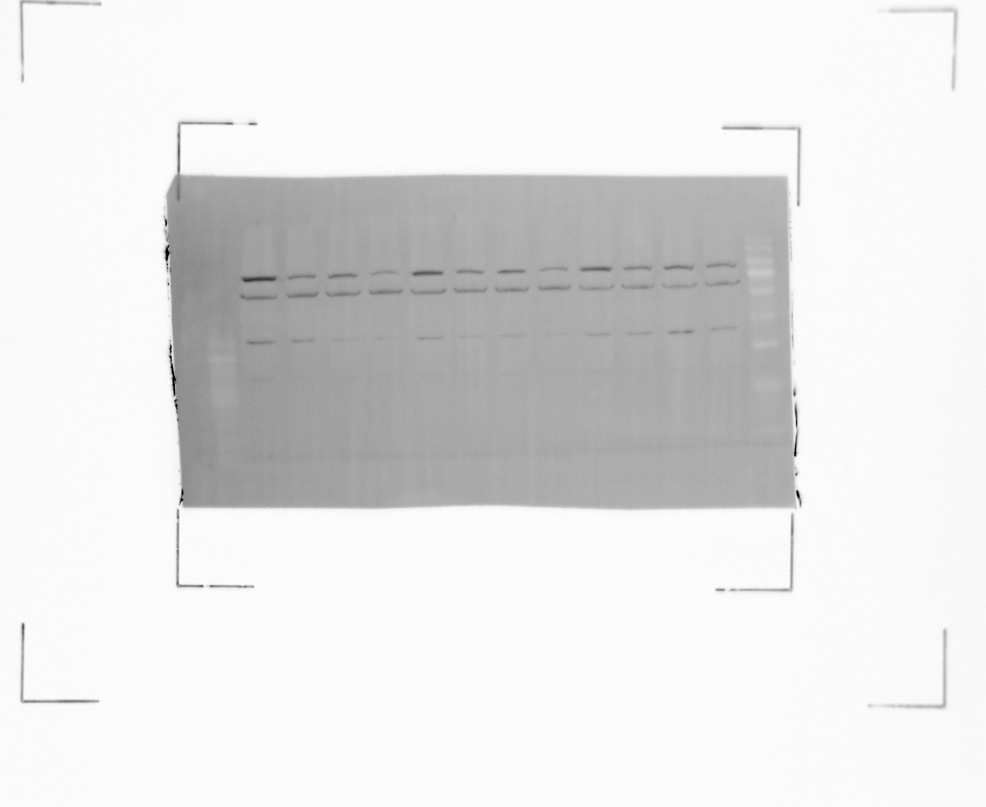
**

-100 kDa

-135 kDa

-63 kDa

-35 kDa

-17 kDa

-180 kDa

-25 kDa

-75 kDa

-48 kDa

SHD+TMZ

Model

TMZ

SHD

SHD+TMZ

Model

TMZ

SHD

SHD+TMZ

Model

TMZ

SHD

**AKT1**

**
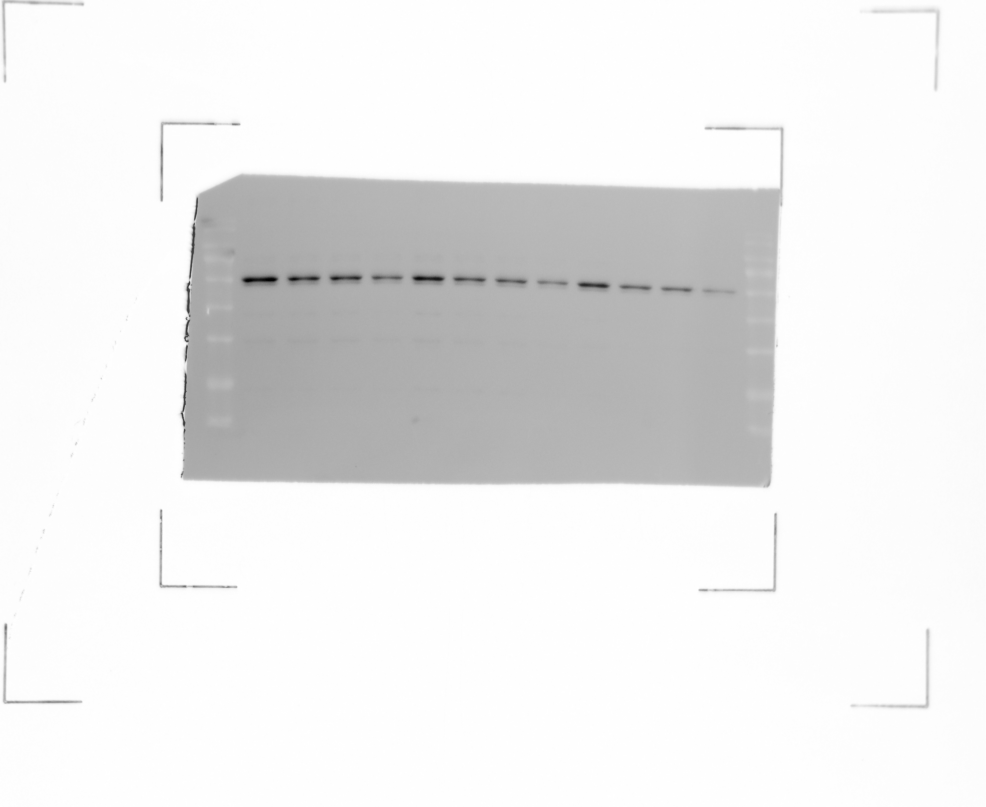
**

SHD+TMZ

Model

TMZ

SHD

SHD+TMZ

Model

TMZ

SHD

SHD+TMZ

Model

TMZ

SHD

**TP53**

**
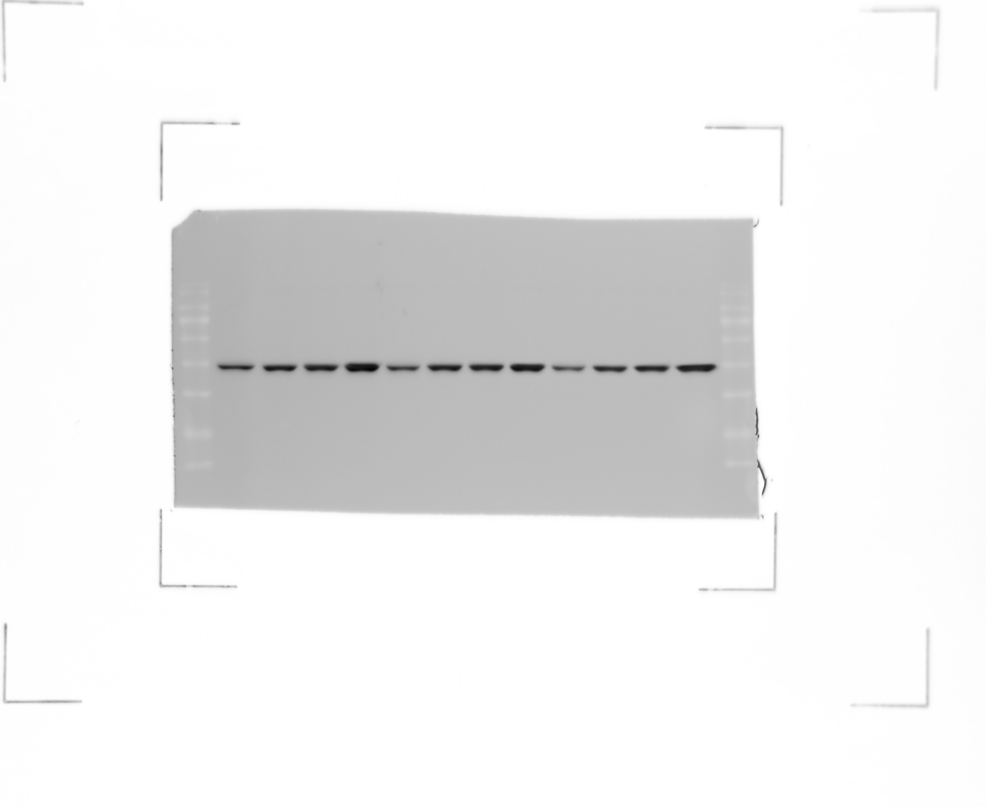
**

SHD+TMZ

Model

TMZ

SHD

SHD+TMZ

Model

TMZ

SHD

SHD+TMZ

Model

TMZ

SHD

**CTNNB1**

**
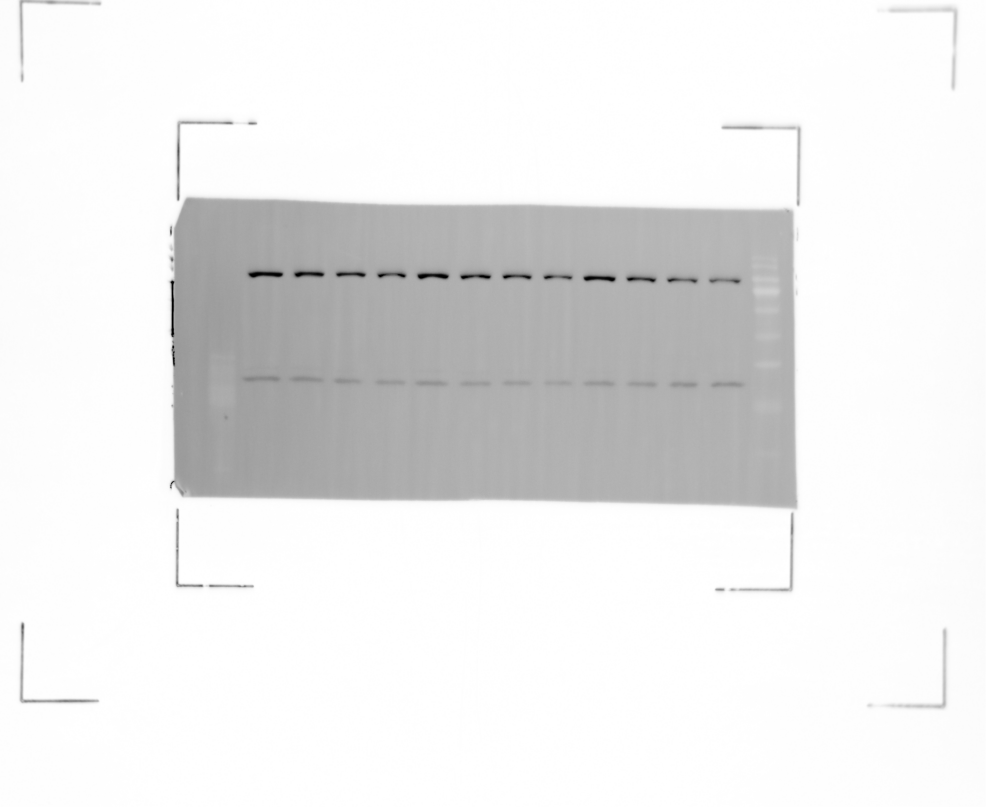
**

-100 kDa

-135 kDa

-63 kDa

-35 kDa

-17 kDa

-180 kDa

-25 kDa

-75 kDa

-48 kDa

SHD+TMZ

Model

TMZ

SHD

SHD+TMZ

Model

TMZ

SHD

SHD+TMZ

Model

TMZ

SHD

**STAT3**

**
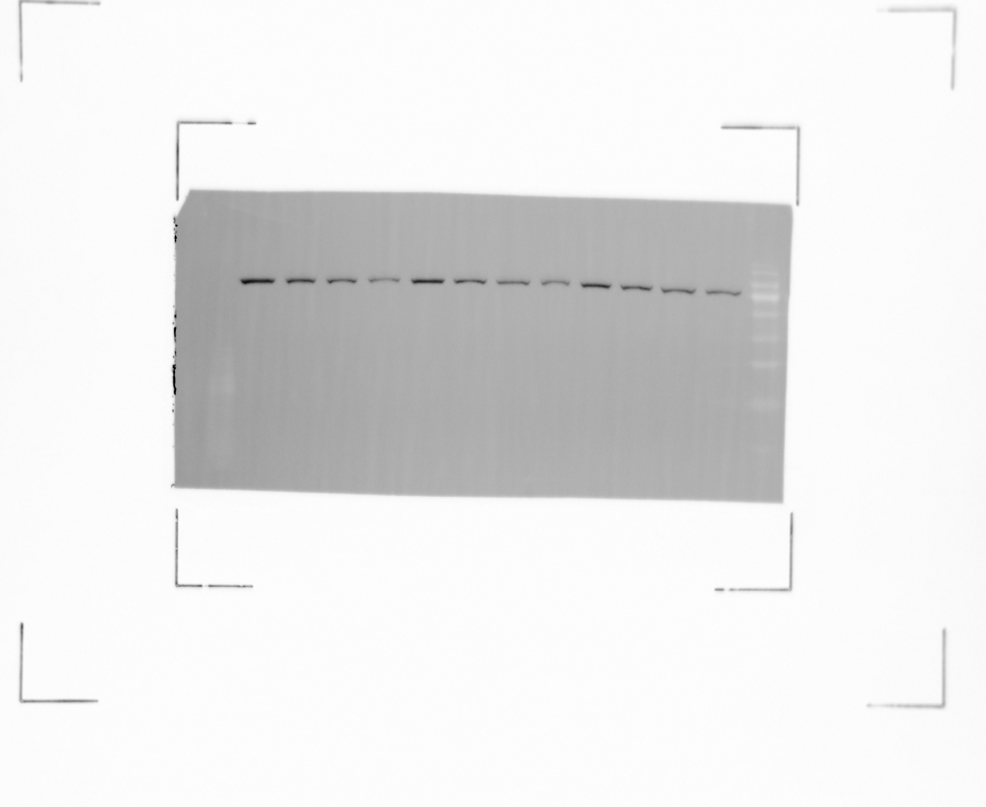
**

SHD+TMZ

Model

TMZ

SHD

SHD+TMZ

Model

TMZ

SHD

SHD+TMZ

Model

TMZ

SHD

**EGFR**

**
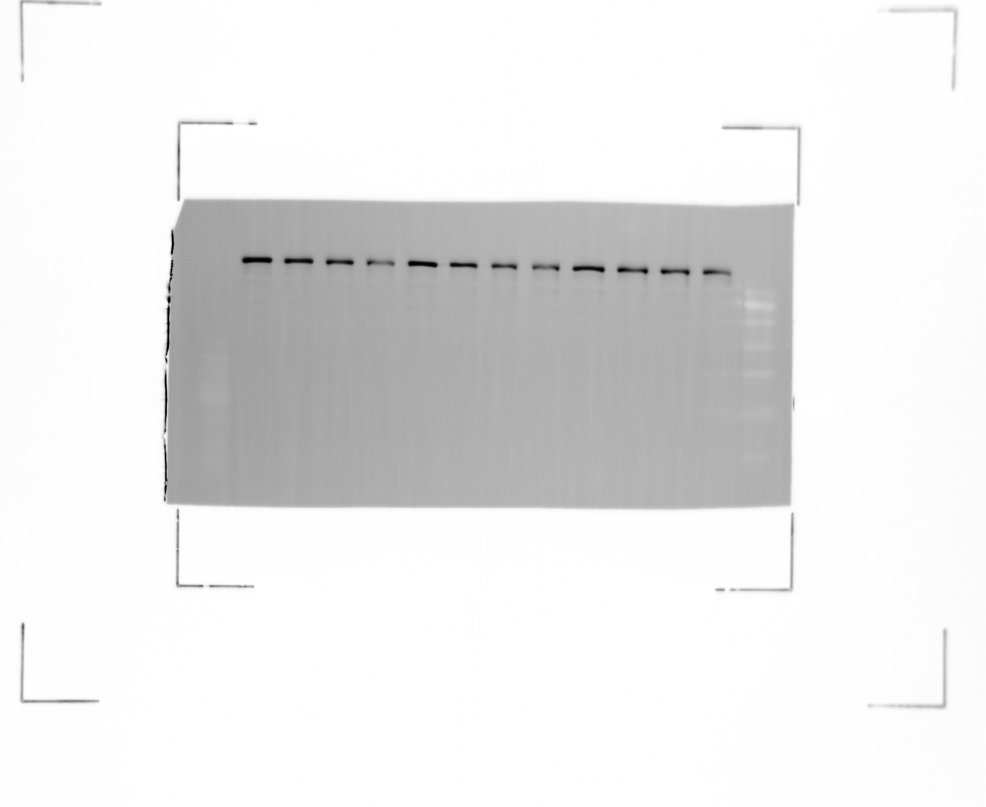
**

SHD+TMZ

Model

TMZ

SHD

SHD+TMZ

Model

TMZ

SHD

SHD+TMZ

Model

TMZ

SHD

**VEGFA**

**
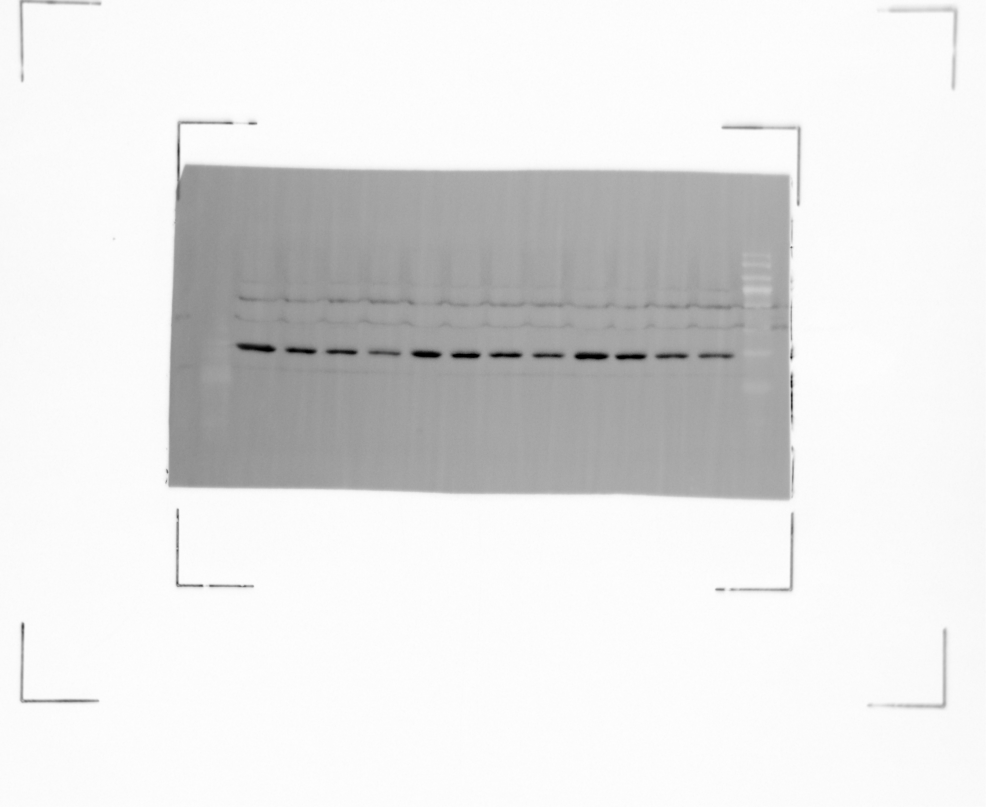
**

SHD+TMZ

Model

TMZ

SHD

SHD+TMZ

Model

TMZ

SHD

SHD+TMZ

Model

TMZ

SHD

**ERBB2**

**
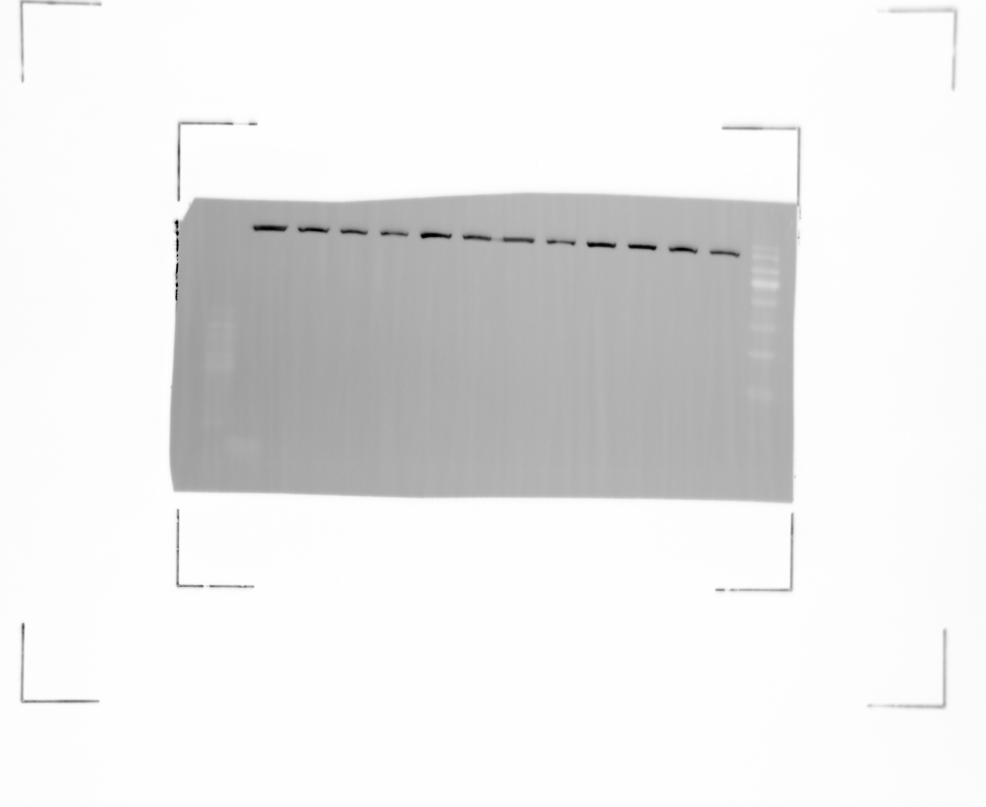
**

SHD+TMZ

Model

TMZ

SHD

SHD+TMZ

Model

TMZ

SHD

SHD+TMZ

Model

TMZ

SHD

**HIF1A**

**
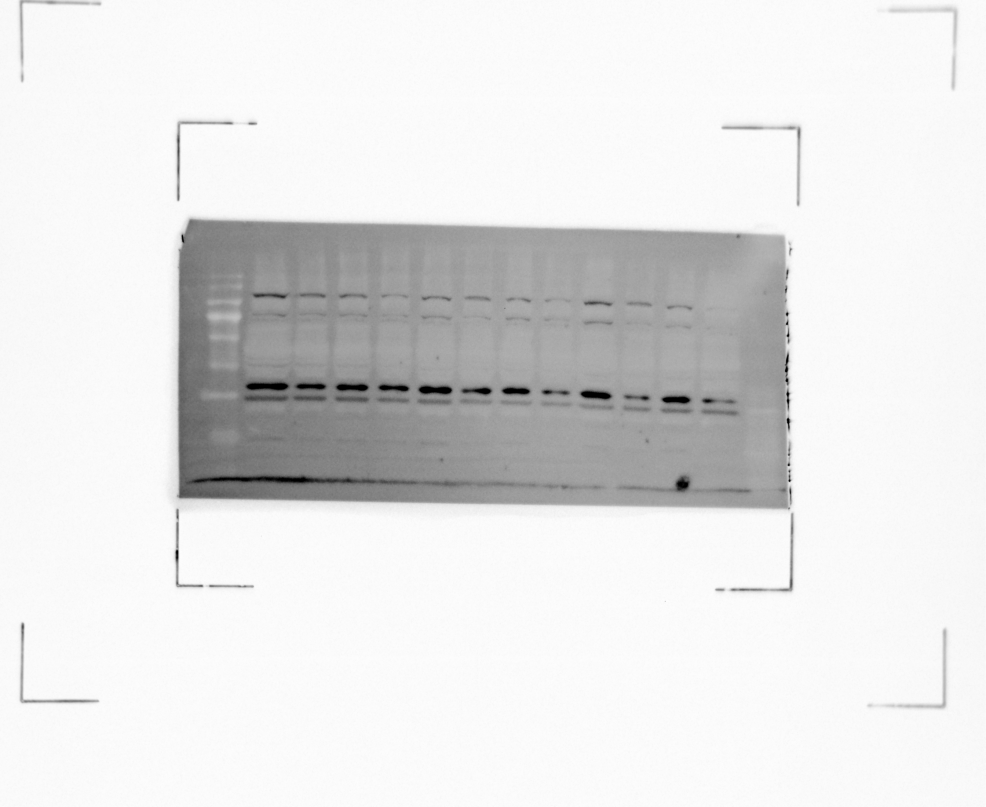
**

SHD+TMZ

Model

TMZ

SHD

SHD+TMZ

Model

TMZ

SHD

SHD+TMZ

Model

TMZ

SHD

**β-actin**

**
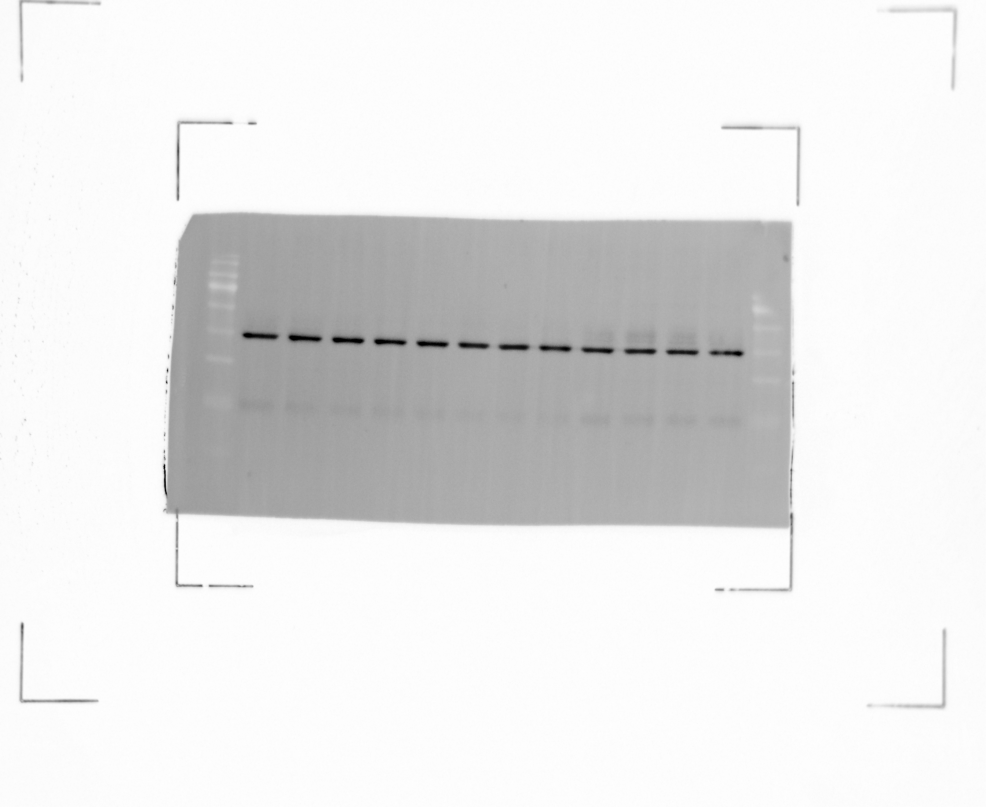
**

SHD+TMZ

Model

TMZ

SHD

SHD+TMZ

Model

TMZ

SHD

SHD+TMZ

Model

TMZ

SHD
